# Chemical profiling analysis of Maca using UHPLC-ESI-Orbitrap MS coupled with UHPLC-ESI-QqQ MS and the neuroprotective study on its active ingredients

**DOI:** 10.1038/srep44660

**Published:** 2017-03-17

**Authors:** Yanyan Zhou, Peng Li, Adelheid Brantner, Hongjie Wang, Xinbin Shu, Jian Yang, Nan Si, Lingyu Han, Haiyu Zhao, Baolin Bian

**Affiliations:** 1 Institute of Chinese Materia Medica, Academy of Chinese Medical Sciences, Beijing, China; 2 Institute of Chinese Medical Sciences, University of Macau, Macau, China; 3 Institute of Pharmaceutical Sciences Pharmacognosy, University of Graz, Graz, Austria; 4 Shandong Rosemed Biopharm LTC, Yanzhou, Shandong province, China.

## Abstract

*Lepidium meyenii* (Maca), originated from Peru, has been cultivated widely in China as a popular health care food. However, the chemical and effective studies of Maca were less in-depth, which restricted its application seriously. To ensure the quality of Maca, a feasible and accurate strategy was established. One hundred and sixty compounds including 30 reference standards were identified in 6 fractions of methanol extract of Maca by UHPLC-ESI-Orbitrap MS. Among them, 15 representative active compounds were simultaneously determined in 17 samples by UHPLC-ESI-QqQ MS. The results suggested that Maca from Yunnan province was the potential substitute for the one from Peru. Meanwhile, the neuroprotective effects of Maca were investigated. Three fractions and two pure compounds showed strong activities in the 1-methyl-4-phenyl-1, 2, 3, 6-tetrahydropyridine (MPTP)-induced zebrafish model. Among them, 80% methanol elution fraction (Fr_5_) showed significant neuroprotective activity, followed by 100% part (Fr_6_). The inhibition of acetylcholinesterase (AChE) and butyrylcholinesterase (BuChE) was a possible mechanism of its neuroprotective effect.

Maca (*Lepdium meyenii*), known as “*Peruvian ginseng*”^1^, has been used as traditional health care food for over 2000 years in South America. According to hypocotyl colors, it was classified in black, purple and yellow varieties^2^. In 1992, Maca was recommended as the safety edible food by Food and Agriculture Organization (FAO). After twenty years of development, it has been considered as one of the star products in the global health care market. Because of its various potential effects, in the early 21^st^ century, Maca was introduced into China successfully and vigorously promoted the cultivation in Yunnan, Xinjiang and Tibet regions at high altitude similar to Peru.

Traditionally, Maca was always used in strengthening body, improving fertility and sexual function. Modern pharmacological studies displayed its effects on depression, rheumatism, premenstrual discomfort and menopausal symptoms^3^. Significantly, along with the increasing risk of neurodegenerative diseases, the neuroprotective effect of Maca has been attracting great concern. The discovery and screening of neuroprotective substances from Maca should be given high priority^4^,^5^,^6^,^7^,^8^,^9^,^10^,^11^,^12^.

Since 2016, the price of Maca decreased dramatically in China. There were a couple of reasons for this. Firstly, a plenty of inferior products of Maca disturbed the market. Secondly, the basic study of Maca could not meet the requirement of market. Both of them came down to the absence of in-depth research of Maca, including the chemical profiling, the reasonable quality standard and the systematic effects evaluation^13^.

Previously, people mainly focused on the nutrient compositions in Maca, such as proteins, amino acids and fatty acids. However, the secondary metabolites were mainly responsible for its multiple functions. The alkaloids, glucosinolates and macaenes should be deserved close attention^14^. UHPLC-ESI-Orbitrap MS was the valid solution for the chemical analysis of secondary metabolites in Maca, which could provide accurate full MS and MS/MS fragments measurements of the target compounds with high sensitivity and precision. For quantitative analysis, UHPLC-ESI-QqQ MS could determine different types of constitutes irrespective of their ultraviolet absorption and the degree of separation by dynamic multiple reaction monitoring (DMRM) method.

In terms of neuroprotective effects evaluation, the zebrafish models of neurodegenerative diseases has been recognized by increasing numbers of researchers^15^,^16^,^17^,^18^,^19^,^20^. Since zebrafish embryos were susceptible to various toxins such as MPTP, which was used as an inducible model of neuronal loss. Transparency was also a unique attribute of zebrafish that permitted direct assessment of drug effects on the nervous system. Also, the other multiple advantages of zebrafish, such as small size, high fecundity and ease of phenotype recognition, made it well suited for high-throughput screening. Meanwhile, in the evaluation of neurodegenerative diseases, MPTP-induced zebrafish model generated a loss of dopaminergic neurons similar to the mid-brain lesion in the Parkinsonian patients^21^.

Generally, in the present study, a sensitive and accurate strategy was developed for the comprehensive chemical analysis of Maca firstly. UHPLC-ESI-Orbitrap MS and UHPLC-ESI-QqQ MS were employed for the qualitative and quantitative analysis respectively. Totally, 160 constituents were detected and identified from 6 fractions of Maca extract. Fifteen of them were selected for the quality control study. Furthermore, the neuroprotective effect of Maca was studied by MPTP-induced zebrafish model with dopaminergic neuronal loss. 80% methanol elution fraction (Fr_5_) showed significant neuroprotective effects, followed by 100% part (Fr_6_). Also, the inhibition of acetylcholinesterase (AChE) and butyrylcholinesterase (BuChE) experiments were performed *in vitro*, which also supported the 80% methanol elution fraction (Fr_5_) as the strongest neuroprotective fraction. Imidazole alkaloids, macamides and macaenes were predominant constituents in Fr_5_. AChE and BuChE were regarded as the potential targets for the neuroprotective effects of Maca.

## Results and Discussion

### The chemical profiling analysis of Maca

**Figure 1:**
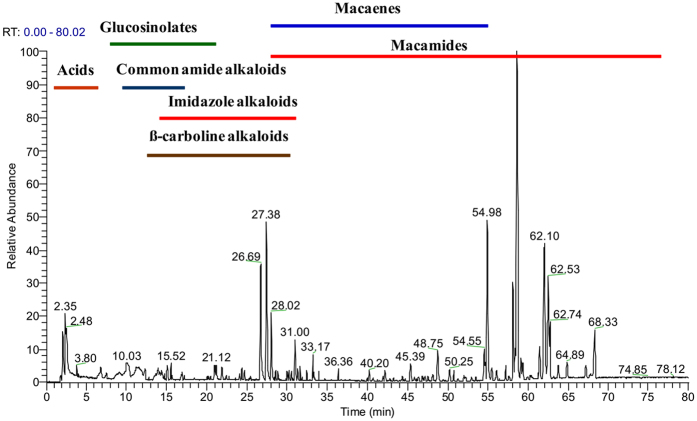
The total ion chromatogram (TIC) of Maca.

The use of Octadecylsilyl gel (ODS gel) remarkably increased chromatographic peaks, and thus achieved the enrichment of minor constitutes. UHPLC-ESI-Orbitrap MS was employed for the analysis. The element compositions of reference standards, unknown compounds and their MS^n^ fragments were calculated by accurate high resolution mass measurements. The total ion chromatogram (TIC) of Maca extract was shown in Fig. 1. In 6 enriched fractions, 160 ingredients were observed and identified, which were separated and enriched specifically according to their polarities. The main components in each fraction was illustrated as follows: Fr_1_ (organic acids), Fr_2_ (glucosinolates), Fr_3_ (β-carboline alkaloids), Fr_4_ (common amide alkaloids, macaridines), Fr_5_ (mainly imidazole alkaloids, macamides, macaenes), Fr_6_ (macamides).

Among them, 30 compounds were identified unambiguously by comparing with the retention time and MS data of reference standards. The other 130 constituents were deduced by their collision induced dissociation pathways together with literatures. Alkaloids, glucosinolates and macaenes were the major chemical ingredients of Maca.

#### Identification of alkaloids in Maca

Macamides, common amide alkaloids, macaridines, β-carboline alkaloids and imidazole alkaloids were five subtypes of characteristic alkaloids in Maca. As we knew, alkaloids always presented high sensetivity in the positive ion mode. Totally, 121 unknown alkaloids were identified.

**Figure 2:**
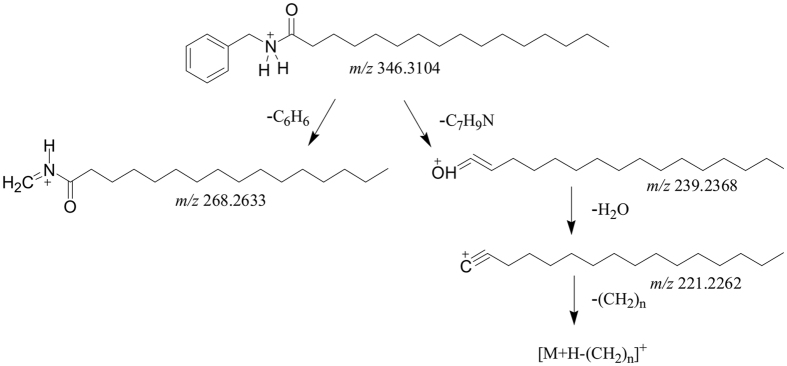
The fragmentation patterns of N-benzylhexadecanamide (No. A_54_).

**Figure 3:**
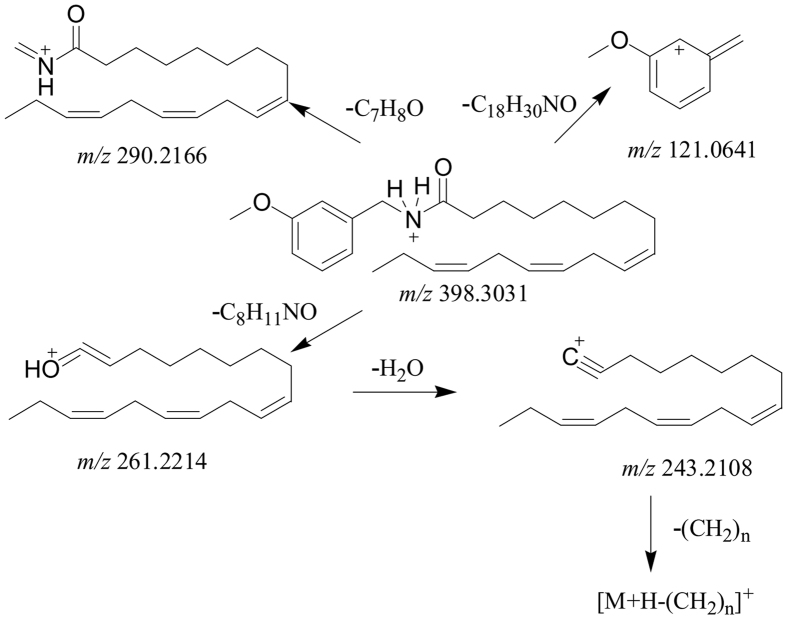
The fragmentation patterns of N-(3-methoxybenzyl)-(9Z, 12Z, 15Z)-octadecatrienamide (No. A_32_).

A_54_ and A_32_ were two representatives of macamides, the fragmentation patterns of which were shown in Figs 2 and 3 respectively. In the MS/MS analysis of them, the diagnostic fragments of A_54_ at *m*/*z* 268.2633 (C_17_H_33_NO) and 239.2368 (C_16_H_30_O) were yielded by neutral loss of phenyl group and the cleavage of amide bond respectively. The fragment ions of A_32_ at *m*/*z* 290.2116 (C_19_H_31_NO) and 261.2214 (C_18_H_28_O) were produced by losing N-3-methoxyl phenyl group and the cleavage of amide bond respectively. The further loss of H_2_O groups from C_16_H_30_O of A_54_ and C_18_H_28_O of A_32_ yielded the product ions at *m*/*z* 221.2265 (C_16_H_28_) and *m*/*z* 243.2093 (C_18_H_26_). Successively, the loss of CH_2_ groups was also monitored in the further MS/MS fragmentations. The MS/MS spectra of A_32_ and A_54_ were shown in Figures S1 and S2 separately. Compound A_58_ (t_R_ = 63.83 min) and A_55_ (t_R_ = 62.23 min) gave [M + H]^+^ ions at *m*/*z* 360.3256 (C_24_H_42_NO) and *m*/*z* 402.3363 (C_26_H_44_NO_2_) in the full mass spectra. In their MS/MS experiments, the fragment ions of A_58_ at *m*/*z* 282.2792 (C_18_H_36_NO) and 253.2419 (C_17_H_33_O) were produced by neutral loss of phenyl group and the cleavage of amide bond respectively. Similarly, the fragment ions of A_55_ at *m*/*z* 294.2792 (C_19_H_36_NO) and 265.2526 (C_18_H_33_O) were yielded by losing N-3-methoxyl phenyl group and the cleavage of amide bond respectively. The fragment at *m*/*z* 235.2419 (C_17_H_31_) of A_58_ and the fragment at *m*/*z* 247.2421 (C_18_H_31_) of A_55_ were generated by the splitting of H_2_O after the cleavage of amide bond. The successive loss of CH_2_ groups was monitored in the further dissociation. The benzene ions were also detected in the MS/MS analysis of two compounds. Thus, A_58_ and A_55_ were identified as N-benzylheptadecanamide (Figure S3) and N-(3-methoxybenzyl)-(9Z)-octadecenamid e (Figure S4). Furthermore, 64 macamides and common alkaloids (A_1_-A_64_) were deduced according to similar fragmentation pathways (Table S1).

**Figure 4:**
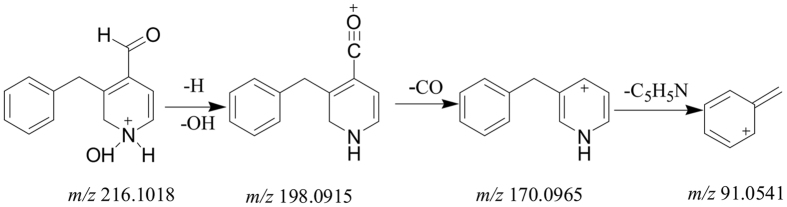
Proposed fragmentation pathways of 3-benzyl-1,2-dihydro-N-hydroxypyridine-4-carbaldehyde (No. A_66_).

As for macaridines, A_66_ showed [M + H]^+^ ion at *m*/*z* 216.1019 (C_13_H_14_NO_2_) in the full mass spectrum. The fragmentation pattern of A_66_ was shown in Fig. 4. The [M + H-H_2_O]^+^ ion at *m*/*z* 198.0915 (C_13_H_14_NO) was firstly determined in the MS/MS experiment. The further neutral loss of CO group generated the fragment ion at *m*/*z* 170.0965 (C_7_H_10_NO_2_). And the fragment ion at *m*/*z* 91.0541 (C_7_H_7_) was yielded by losing C_5_H_5_N group.

The ESI-MS spectrum of A_65_ (t_R_ = 24.33 min) showed a [M + H]^+^ ion at *m*/*z* 218.1175 (C_13_H_16_NO_2_). The loss of H_2_O group yielded [M + H-H_2_O]^+^ ion at *m*/*z* 200.1279 (C_13_H_14_NO). The [M + H-C_6_H_5_]^+^ and [M + H-C_6_H_9_NO_2_]^+^ ions at *m*/*z* 140.0708 ions (C_7_H_10_NO_2_) and 91.0541 (C_7_H_7_) were found in the MS/MS spectrum, indicating that the further neutral loss of phenyl group and the existence of benzyl group. Thus, A_65_ was deduced as 3-benzyl-1, 2-dihydro-N-hydroxypyridine-4-methoxy (Figure S5). The other 2 macaridines (A_66_, A_67_) were identified following the same dissociation pathways (Table S2).

Besides, the MS dissociation of some characteristic β-carboline alkaloids and imidazole alkaloids such as (1 R, 3 S)-1-methyltetrahydro-β-carboline-3-carboxylic acid (A_110_) and 1, 3-dibenzyl-4, 5-dimethylimidazolium (A_82_) were investigated as well. A_82_ showed the representative fragments [M + H-benzyl]^+^ and [benzyl]^+^ at *m*/*z* 185.1075 and *m*/*z* 91.0541 according to the literature^22^. In line with the mass data and literatures^23^,^24^, the cleavage of NH_3_, C_2_H_2_, C_2_O_2_, C_2_H_4_ and CH_2_ groups from the precursor ion of A_110_ was detected.

In the MS/MS analysis of A_84_ (t_R_ = 28.84 min), the [M + H]^+^ ion at *m*/*z* 305.2011 (C_21_H_25_N_2_) was detected. The [M + H-C_7_H_7_]^+^ and C_7_H_7_^+^ ions were also found at *m*/*z* 213.1390 (C_14_H_17_N_2_) and 91.0542 (C_7_H_7_). Besides, two fragments at *m*/*z* 199.12318 (C_13_H_15_N_2_) and *m*/*z* 185.1074 (C_12_H_13_N_2_) were assigned as the successive loss of CH_2_ moieties. All the fragmentation pattern of A_84_ revealed itself to be 1, 3-dibenzyl-2-ethyl-4, 5-dimethylimidazilium (Figure S6). The MS behaviors of 38 imidazole alkaloids (A_68_-A_105_) were in line with that of A_84_ (Table S3).

The ESI-MS spectrum of A_111_ (t_R_ = 18.21 min) showed a [M + H]^+^ ion at *m*/*z* 245.1284 (C_14_H_17_N_2_O_2_). In the MS/MS fragmentation, [M + H-H_2_O]^+^ ion at *m*/*z* 227.1177 (C_14_H_15_N_2_O) was detected. Another two fragments at *m*/*z* 167.0813 (C_8_H_11_N_2_O_2_) and *m*/*z* 153.0658 (C_7_H_9_N_2_O_2_) were generated from the neural loss of phenyl and benzyl group from [M + H]^+^ ion respectively, which suggested that A_111_ was (1R, 3S)-1-ethyltetrahydro-β-5,6-carboline-3-carboxylic acid (Figure S7). Sixteen β-carboline alkaloids (A_106_-A_121_) were also assigned following the same dissociation pathways (Table S4).

#### Identification of glucosinolates and acids in Maca

**Figure 5:**
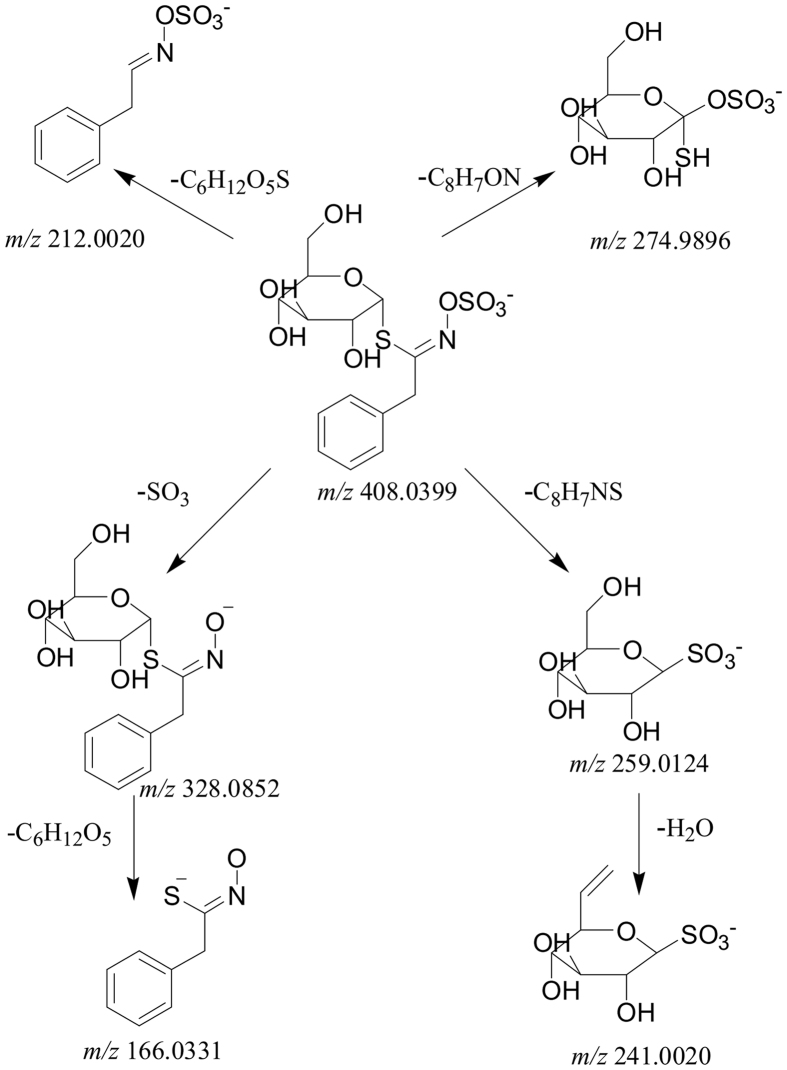
The fragmentation patterns of benzylglucosinolate (No. G_5_).

Glucosinolates and acids displayed the [M-H]^−^ ions with sufficient abundance in the negative ion mode. In agreement with literatures, the cleavage of SO_3_, glucose, C_8_H_7_ON, C_8_H_7_NS, C_6_H_12_O_5_S and H_2_O groups was detected in the MS/MS spectra^25^,^26^. Take G_5_ as a reference compound for example (Fig. 5), the ESI-MS spectrum of G_5_ (t_R_ = 14.96 min) showed a [M-H]^−^ ion at *m*/*z* 408.0423 (C_14_H_19_O_9_NS_2_). The [M-H-C_7_H_7_-N = C = S]^−^ ion at *m*/*z* 259.0124 (C_6_H_12_O_9_S) was observed in the MS/MS experiment. The further loss of H_2_O from this obtained ion at *m*/*z* 241.0020 (C_6_H_10_O_8_S) and the [M-H-C_7_H_7_-N = C = O]^−^ ion at *m*/*z* 274.9896 (C_6_H_12_O_8_S_2_) were detected as well. All these three aforementioned ions were formed through intramolecular rearrangements, in which the sulfate group was transferred to the thioglucose moiety. Moreover, the ion at *m*/*z* 212.0020 (C_8_H_7_O_4_NS) was deduced as the loss of the D-thioglucose group from [M-H]^−^ ion. The other two fragments at *m*/*z* 328.0852 (C_14_H_19_O_6_NS) and 166.0331 (C_8_H_7_ONS) were assigned as the loss of SO_3_ (80 Da) and further loss of glucose^26^. Additionally, the loss of H_2_O group was considered as the representative fragmentation pathway in acids (Table S5).

In the negative ion mode, 14 glucosinolates (Table S6) were detected and identified. Among them, 3 glucosinolates (G_3_, G_5_, G_8_) and 5 acids (C_1_-C_5_) were isolated and characterized as reference standards.

G_11_ (t_R_ = 21.30 min) was a classical compound with dominant amount, which showed [M-H]^−^ ion at *m*/*z* 450.0504 (C_16_H_20_O_10_NS_2_). The MS/MS spectrum produced [M-H-Glc]^−^ ion at *m*/*z* 287.0550 (C_10_H_9_O_5_NS_2_). The [M-H-SO_3_]^−^ ion at *m*/*z* 370.0949 (C_16_H_20_O_7_NS) and the [M-H-C_8_H_7_ON]^−^ ion at *m*/*z* 316.9993 (C_8_H_13_O_9_S_2_) were also found. The cleavages of C_8_H_7_NS from [M-H]^−^ ion was detected at *m*/*z* 301.0225 (C_8_H_13_O_10_S). The further loss of H_2_O group generated the fragment at *m*/*z* 283.0117 (C_8_H_11_O_9_S). Meanwhile, the [M-H-C_8_H_12_O_5_S]^−^ and [M-H-C_8_H_12_O_5_S-H_2_O]^−^ fragments at *m*/*z* 230.0121 (C_8_H_8_O_5_NS) and *m*/*z* 212.0017 (C_8_H_6_O_4_NS) were also observed. The product ion at *m*/*z* 166.0328 (C_8_H_8_ONS) were attributable to the breakage of C_8_H_12_O_6_ group from C_16_H_20_O_7_NS. All of the fragmentation patterns revealed G_11_ as acetyl-benzylglucosinolate (Figure S8).

#### Identification of macaenes in Maca

As reported before, macaenes were also the characteristic compounds from Maca^27^. In the present study, 11 macaenes were observed in the positive ion mode, among of which, 5-oxo-6E, 8E-octadecadienoic acid (M_7_) has high content. M_7_ demonstrated obvious fragments at *m*/*z* 277.2165 and *m*/*z* 259.2059, indicating the continuous loss of 2H_2_O groups.

M_9_ (t_R_ = 53.02 min) exhibited [M + H]^+^ ion at *m*/*z* 309.2418 (C_19_H_33_O_3_) in full scan mass spectrum. Its MS/MS spectrum presented [M + H-H_2_O]^+^ fragment at *m*/*z* 291.2321 (C_19_H_31_O_2_). The fragments of this obtained ion at *m*/*z* 277.2161 (C_18_H_29_O_2_) and *m*/*z* 153.0911 (C_9_H_13_O_2_) indicated the loss of CH_2_ and C_10_H_18_ groups respectively. The fragment at *m*/*z* 125.0962 (C_8_H_13_O) was assigned as the cleavage of CO from C_9_H_13_O_2_ group. In addition, the product ion at *m*/*z* 185.1174 (C_10_H_17_O_3_) was detected clearly, which was attributed as the loss of C_9_H_16_ group from [M + H]^+^ ion. Consequently, M_9_ was putatively characterized as 5-oxo-6E, 8E-nineteencadienoic acid (Figure S9). The MS behaviors of M_1_-M_11_ were in line with that of M_9_ (Table S7).

#### Identification of other compounds in Maca

Nine other compounds were detected and identified including flavonoids, organic esters, pyridine and benzylcyanide constitutes (Table S8). For example, O_6_ showed the loss of C_2_H_4_, C_3_H_6_, and C_9_H_10_O_2_ clearly in the MS/MS spectrum, which was in accordance with Licochalcone A^28^.

### Quantitative analysis of samples

Ion polarity switching mode was adopted in the quantitative analysis. Glucosinolates and acids were determined in the negative ion mode, while others were detected in the positive ion mode.

### Method validation

#### Linearity of calibration curves, limit of detection (LOD) and limit of quantification (LOQ)

The internal standard method was employed to calculate the contents of 15 compounds in Maca. The standard solutions with internal standard were diluted with methanol to six different concentrations for the construction of calibration curves. The ratio of peak area to internal standard (Yi/Ys) of each analyte was plotted against the injection concentration (X, ng.mL^−1^). All the calibration curves indicated good linearity with determination coefficients (r) from 0.9951 to 0.9998. The limits of detection (LOD) and the limits of quantification (LOQ) were evaluated at a signal-to-noise ratio (S/N) of 3/1 and 10/1 respectively. The parameters of LOD and LOQ for each constituent in this experiment were from 0.05~164.95 ng.mL^−1^ and 0.05~824.74 ng.mL^−1^ (shown in Table 1 29,30).

#### Precision, stability and repeatability

The intra-day and inter-day precisions of the present method were calculated by analyzing the standard solution under the optimized experimental conditions. The RSD values of them were 0.41%~2.46% and 0.43%~2.71%. The RSD values of stability of each constituent in 48 hours at room temperature (n = 6) were 1.01%~2.84%. Furthermore, the sample solutions for Maca were prepared in parallel (n = 6) to evaluate the repeatability and achieved the RSD of 1.02%~2.63% (Table S9).

#### Recovery

The recovery was used to evaluate the accuracy of the method. Nine copies of 1 g Maca were taken for recovery test. The mixed standard solutions of 15 constituents were added according to three levels (1:0.8, 1:1, 1:1.2) respectively. The mixtures were treated as the preparation procedure of sample and analyzed using the method described above. Recovery (R) was calculated as R = 100 (Mmeasured−Minitial)/Madded (Mmeasured = measured amount in the recovery sample, Minitial = initial amount in the sample, Madded = amount in the standard solution used) for each compound. The average recovery rate of each constituent was 96.27%~98.89%, with the RSD values from 0.60% to 3.11% (Table S10), which met the requirements for the determination of 15 constituents in Maca.

#### Analysis of samples

**Figure 6:**
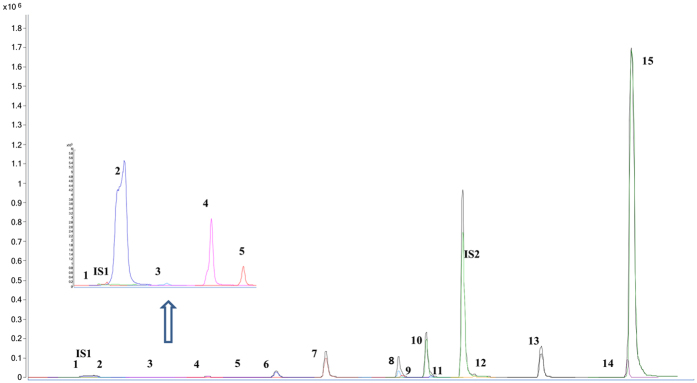
The MRM chromatogram of mixed compounds (15 markers and 2 internal standards). 1.C_3_;**2.**C_5_;**3**G_8_;**4.**G_5_;**5.**G_3_;**6.**A_4_;**7.**A_5_;**8.**A_66_;**9.**A_67_;**10.**A_8_;**11.**A_121_; **12.**O_6_;**13.**O_9_; **14.**A_52_; **15.**A_54_. (**A**) **1–5**: negative-DMRM node; (**B**). **6–15**: positive-DMRM mode.

This validated UHPLC-ESI-QqQ MS method was used for the quantification analysis of 15 constituents in 17 batches of Maca under the DMRM mode (Fig. 6). The analysis time was shortened to 15 minutes. Each constituent was calculated by their respective calibration curve, and the quantification results were shown in Table 2.

15 markers were identified unambiguously by comparing the retention times and transitions in DMRM mode of reference standards. Two internal standards were employed to guarantee the accuracy of determination. The polarity switching mode of QqQ MS was used to achieve the highest response intensities of each constituent. The quantification of Licochalcone A and 3-benzyl-1, 2-dihydro-N-hydroxypyridine-4-carbaldehyde was investigated as well, which were two representative constituents but with low contents in Maca.

The total amounts of investigated 15 compounds in Maca varied from 1.20% to 8.12%. Among them, glucosinolates (10.97~79.84 mg.g^−1^) and alkaloids (0.54~2.99 mg.g^−1^) were the predominant constituents. The contents of glucosinolates were significantly more than the other markers.

Significant variations were observed in different alkaloids. The contents of macamides were the highest with the amounts from 0.54 to 2.95 mg.g^−1^. Macamides were reported as one of the important secondary metabolites of Maca with neuroprotective, anti-fatigue, improving fertility and sexual functions effects. What’s more, β-carboline alkaloids (A_121_) and macaridines (A_66_, A_67_) were also detected in present analysis, the contents of which were 0~1.6 mg.g^−1^ and 1.47~72.61 mg.g^−1^. β-carboline alkaloids displayed neuroprotective effect^24^.

In general, glucosinolates and β-carboline alkaloids in the samples from Peru were higher than those cultivated in China. However, the contents of organic acids, macaridines, common amide alkaloids, macamides in Maca from China were higher than or similar to that from Peru. The results indicated that Maca cultivated in China especially in Yunnan province could be used as the potential substitute.

Among the Chinese samples, the contents of secondary metabolites in Yunnan province (Sample 7-Sample 17) were higher than those from the other two origins. The contents of effective constitutes in the samples from Xinjiang province (Sample1and Sample 2) were relatively lower. Generally, the content tendency of different types constitutes in Maca was similar to each other. Glucosinolates (G_3_, G_5_, G_8_) and macamides (A_52_, A_54_) presented large amount (glucosinolates were 8~89 times than macamides), followed by organic acids (C_3_, C_5_), macaridines (A_66_, A_67_), common amide alkaloids (A_4_, A_5_), Licochalcone A (O_6_).

In addition, Maca was always classified according to different hypocotyls colors in the market. In present study, a yellow and a purple Maca samples from the same growing environment and growth cycle were included in the analysis. Interestingly, the content of glucosinolates in 15 yellow Maca samples (Samples 1–5. 8–17) was higher than that in 2 purple Maca samples (Samples 6–7). However, from the data we collected, the types and contents of ingredients in purple and yellow Maca samples had no significant difference with each other, which was also in accordance with the literature^31^. Accurately, the geographical origin played a more important role than color. The results indicated that the contents of secondary metabolites in yellow Maca was in line with those in purple Maca generally. The quality discrepancy of them was not as significant as propagandized in market.

Also, the main components in different parts of Maca (roots and up-ground parts) were studied preliminary as well. The contents of active ingredients in the roots were higher than those in leaves. Three experimental samples were collected from the same Maca plant (Sample 12 and Leaves of Maca). To the best of our knowledge, in the biosynthetic pathways of macamides, benzylglucosinolate, free linoleic and linolenic acids, benzyl isothiocyanate and benzylamine were direct or indirect precursors^32^,^33^. The accumulation of macamides was significantly associated with the contents of fatty acid and benzylamine. Thus, one of the important quality criterions of Maca was the levels of macamides and glucosinolates. In present study, both of them were higher in roots than those in leaves. The up-ground parts were regarded as the potential resources for the enrichment of the effective macamides.

In the cultivation of Maca, the harvest time, longitude and altitude affected its quality greatly. The present investigation provided a valuable strategy for the quality evaluation of Maca from their chemical profiling no matter the samples from different regions and varieties.

### Neuroprotective effect screening of active fractions and pure compounds

**Figure 7:**
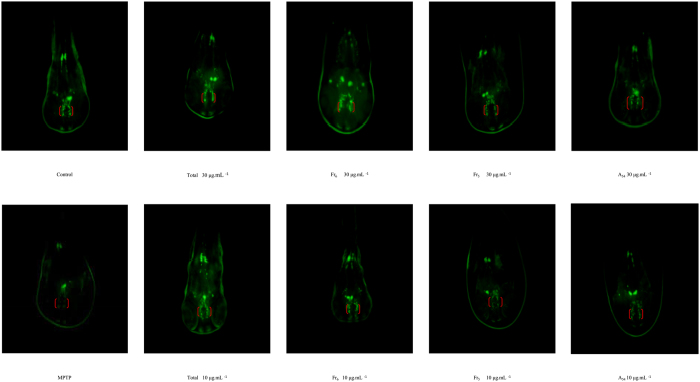
Representative pictures of Maca fractions and pure compounds against the dopaminergic neuron loss treated with MPTP in Zebrafish. DA neurons were examined in zebrafish by whole-mount immunostaining with an antibody against TH, the rate-determining enzyme involved in the synthesis of DA. Thus, TH^+^ neurons in the diencephalic area of the zebrafish brain were considered as DA neurons.

**Figure 8:**
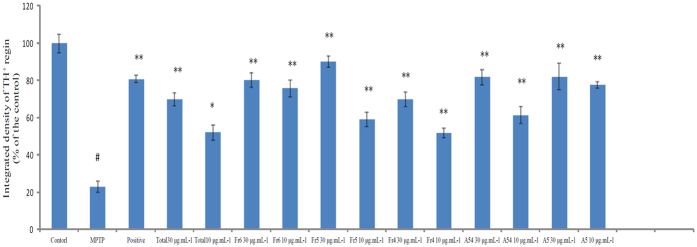
The quantitative results of Maca fractions and pure compounds against the dopaminergic neuron loss treated with MPTP in Zebrafish. Quantitative analysis of TH + neurons in zebrafish brain. Data are expressed as a percentage of the control group. Each bar represents mean ± SD. ^#^p < 0.05 vs. the control group; *p < 0.05 vs. and **p < 0.01 vs. the MPTP-treated group.

Although, Maca has displayed the neuroprotective activity both *in vivo* and *in vitro*, its corresponding bioactive components and possible mechanism were still not clearly^8^,^9^,^34^. In present study, the zebrafish embryos were treated with MPTP to form the DA neuronal loss model, which was always used for the neuroprotective evaluation. The DA neuronal loss site of zebrafish was located in the ventral diencephalic clusters (indicated by red brackets) (Fig. 7). As a result, by compared with the controls, MPTP model group resulted in 70% reduction of TH-positive neurons in the diencephalic area of the zebrafish embryo. The total Maca methanol extract displayed neuroprotective effect against MPTP-induced toxicity in zebrafish. Then, the activity evaluation of 6 fractions enriched by ODS column and 2 pure compounds was examined. The fractions and pure compounds were able to inhibit DA neuron loss by approximately 30~60% compared with the MPTP group. Consequently, Fr_4_-Fr_6_ could increase the amount of dopamine neurons in different degrees with dose-dependent effect, especially for 80% methanol elution fraction (Fr_5_) (Fig. 8). However, Fr_1_-Fr_3_ showed no or little corresponding neuroprotective effects. The LC-MS chromatograms of Fr_4_-Fr_6_ were shown in Figures S10–S12.

N-benzylhexadecanamide (A_54_) and N-acetylbenzylamine (A_5_) were two important active secondary metabolites in Maca with higher abundance. Because of their relative higher contents, A_5_ and A_54_ were easily to be isolated from the plant. Thus, A_5_ and A_54_ were chosen as the representative ingredients for the neuroprotective effect evaluation. Combination with chemical profiling analysis, common alkaloids and macaridines were predominant constitutes in 50% methanol elution fraction (Fr_4_). 80% methanol elution fraction (Fr_5_) mainly contained imidazole alkaloids, macamides and macaenes. Macamides were also observed in 100% methanol elution fraction (Fr_6_). Interestingly, both Fr_5_ and Fr_6_ contained N-benzylhexadecanamide (A_54_) with high abundance. Therefore, A_54_ was selected as the representative pure compound for the effective confirmatory experiment. As the results, at the dose of 30 and 10 μg mL^−1^, A_54_ could significantly prevented the MPTP-induced decrease in TH^+^ region area, and showed neuroprotective actions in a dose-dependent manner. Meanwhile, N-acetylbenzylamine (A_5_) demonstrated noteworthy protections against MPTP-induced toxicity with dose-dependent manner, which was the major constituent in Fr_4_ fraction. The results were in accordance with the neuroprotective properties of the pentane extract of Maca reported before. Macamides, a class of benzylated and 3-methoxylbenzylated alkamides, were identified as the major characteristic effective compounds of Maca^35^. Because of its excellent solubility profile, macamides could act on the endocannabinoid system and showed fatty acid amide hydrolase (FAAH) inhibitory activities^5^. In present study, A_54_ also showed dose-dependent neuroprotective effect by restoring dopaminergic neuronal loss. The results inspired that Maca was available for the impairment of the endocannabinoid system in dopaminergic neurons, which was closely related to a serious of neurodegenerative diseases, such as Alzheimer’s disease, Parkinson’s disease and so on. Additional studies of macamides should be carried out on the mechanism of FAAH inhibition involving the endocannabinoid system^36^. Further, macaridines, imidazole alkaloids and macaenes in Maca also should be concerned. Additionally, the results also demonstrated that zebrafish could be useful for the neuroprotective activity evaluation. The brain of zebrafish embryonic contained clusters of dopaminergic with the characteristics of inexpensive, low-maintenance and abundantly produce offspring. Furthermore, a significant inherent advantage was its transparency. Therefore, zebrafish could provide invaluable insights for large scale screening, drug discovery, modeling behavioral and functional parameters of neurodegeneration disorders and preclinical treatments^37^,^38^,^39^,^40^,^41^,^42^,^43^.

Parkinson’s disease (PD) was a progressive neurodegenerative disorder characterized by the selective loss of nigral dopaminergic neurons and a reduction in striatal dopaminergic fibers^44^. MPTP was metabolized to MPP^+^, which generated a similar loss of dopaminergic neurons with corresponding Parkinsonian symptoms^45^. Thus, we used zebrafish treated with MPTP as the neuronal loss model. Meanwhile, as reported in patients with Parkinson’s disease, both the dopaminergic and cholinergic systems underwent degeneration, which led to deficits in dopamine and acetylcholine at synapses. As for the cholinergic system, MPTP also decreased the gene expression of choline acetyltransferase (ChAT) while increased the expression of acetylcholinesterase (AChE)^46^. In present study, we attempted to bring new evidence supporting the potential neuroprotective action of Maca in PD, focusing on the interaction between dopaminergic and cholinergic systems. Dopamine was supposed to possess modulatory effect in cholinergic transmission, which played a critical role in modulating cortical cholinergic activity by GABAergic intracortical circuits^46^. And the dopaminergic system might be dysfunctional in AD, which was possibly generated by the disrupting of the cholinergic system^47^,^48^. Thus, after the evaluation of Maca on dopamine neuronal loss model in zebrafish by the method of *anti-tyrosine hydroxylase (TH) whole-mount immunostaining*, the AChE and BuChE inhibition activities of 6 fractions and pure compound were carried out. Collectively, these findings indicated that Maca showed neuroprotective activity through the synergistic effect of dopaminergic system and cholinergic system, which needed to be further validation.

Another important reason for the analysis of AChE and BuChE inhibition was from the structures of the neuroprotective chemicals. As we all know, the structural similarities of endocannabinoids and macamides indicated their potential neuroprotection effects. The purified macamides or its synthetic derivatives suggested highly possible activities on the endocannabinoid system^49^. As we knew, the impairment of the endocannabinoid system in dopaminergic neurons would result in many neurological and psychiatric disorders such as Alzheimer’s disease, Parkinson’s disease, depression and schizophrenia^50^,^51^. Meanwhile, dual FAAH/ChE inhibitors, with well-balanced nanomolar activities might be considered as new promising candidates for Alzheimer’s disease treatment, which also suggested the close relationship between endocannabinoid system and cholinergic system^52^. This possible collaboration between cholinergic and dopaminergic neurotransmission in the midbrain raised the possibility of targeting both systems simultaneously to treat PD and AD in the future. Therefore, the preliminary analysis of AChE and BuChE were included in the manuscript. The *in vitro* AChE and BuChE inhibition assays showed (Table 3) that Fr_4_ (IC_50_ = 50.78 μg.mL^−1^), Fr_5_ (IC_50_ = 5.37 μg.mL^−1^) and Fr_6_ (IC_50_ = 15.77 μg.mL^−1^) displayed significant AChE inhibitory activity. Similarly, Fr_4_ (IC_50_ = 45.11 μg.mL^−1^), Fr_5_ (IC_50_ = 5.41 μg.mL^−1^) and Fr_6_ (IC_50_ = 23.39 μg.mL^−1^) showed significant BuChE inhibitory activity. The results were in accordance with those *in vivo* experiments. As the mutual constitute in Fr_5_ and Fr_6_, N-benzylhexadecanamide (A_54_) was selected to validate this mechanism as pure compound, which also displayed high AChE (IC_50_ = 14.23 μg.mL^−1^) and BuChE (IC_50_ = 17.54 μg.mL^−1^) inhibitory activities. What’s more, A_54_ were reported as dual AChE/BuChE inhibitors without remarkable side effects^53^,^54^,^55^. In conclusion, Fr_5_ and macamides produced the healing efficacy by increasing the acetylcholine and butyrylcholine level in brain.

## Conclusions

In this paper, a high sensitive and efficient strategy for the integrating quality control of Maca was established. Alkaloids, glucosinolates, and macaenes were detected as the predominant secondary metabolites in this plant. Among them, five types of alkaloids were observed, including macamides, imidazole alkaloids, macaridine, β-carboline alkaloids and common amide alkaloids. According to the quantitative analysis of 15 major chemical markers in Maca, the contents variety of different origins, crust colors and different parts of plant were clarified. Meanwhile, the neuroprotective effects of 6 fractions against MPTP induced neurotoxicity zebrafish model were examined. 80% methanol elution fraction (Fr_5_) and 100% methanol elution fraction (Fr_6_) were regarded as the most effective neuroprotective parts. Both of these two parts contained macamides. The verification test by pure compounds also proved that alkaloids were the neuroprotective constituents in Maca. The inhibition of AChE and BuChE was one of the possible mechanisms. These findings suggested that Maca was a valuable health care food for the neurodegeneration disease such as Alzheimer Disease, Parkinson’s desease and so on.

## Methods

### Chemicals and Reagents

Methanol for extraction was obtained from Honeywell Burdick and Jackson (Swedesboro, NJ, USA). HPLC grade acetonitrile was purchased from Merck Company (Rahway, NJ, USA). HPLC grade formic acid was provided by ROE Scientific Inc. (Delaware, USA). MPTP, Dimethyl Sulphoxide (DMSO), Trizma, 5,5′-Dithiobis (2-nitro-benzoic acid), Acetylthiocholine iodide (ATCI), AChE from Electophorus electricus electric eel Type VI-S (AChE), S-Butyrylthiocholine iodide (BTCI), BuChE from equine serum (BuChE), Physostigmine (eserine) were purchased from Sigma-Aldrich (St. Louis, MO, USA). All other chemicals and reagents were of high analytical grade.

The reference standards were isolated and purified by authors, including m-methoxybenzylglucosinolate(G_3_), benzylglucosinolate(G_5_), p-hydroxybenzylglucosinolate(G_8_), N-(3-methoxybenzyl)-hexadecanamide(A_52_), N-benzylbenzamide(A_8_), N-benzylhexadecanamide(A_54_), LicochalconeA(O_6_), 3-benzyl-1,2-dihydro-N-hydroxypyridine-4-carbaldehyde(A_66_), (3-methoxybenzyl)–N-pyridine-4-carbaldehyde(A_67_), N-acetylbenzylamine (A_5_), 2-phenylacetamide(A_4_), Nicotinic acid(C_3_), Succinic acid(C_5_), (1R, 3S)-1-methyl-β-carboline-3-carbaldehyde(A_121_), Dibutyl phthalate(O_9_). Silica gel, RP-C_18_ (200–300 mesh, Qingdao Marine Chemical Factory, Qingdao, China), sephadex LH-20 column chromatography and preparation liquid chromatography were used in the isolation. Their structures were identified by MS, NMR and UV spectra (Figure S13). 4-Aminohippuric acid (IS_1_), Evodiamine(IS_2_) were employed as the internal standards, which were purchased from the National Institute for the Control of Pharmaceutical and Biological Products (Beijing, China). The purities of all the reference standards were over 95%.

### UHPLC-ESI-Orbitrap MS analysis and UHPLC-ESI-QqQ MS analysis

#### Preparation of samples solutions

3 g powder of Maca was extracted under ultrasonic in 30 mL methanol for 30 min. After centrifugation at 12000 g for 10 min, the supernatant was condensed to 2 mL, and applied to an Octadecylsilyl gel column (ODS, 200 mL, 3.5 cm × 60 cm) eluted with 5%, 10%, 30%, 50%, 80%, 100% (800 mL) methanol. Each 800 mL of the elution was collected as one fraction^56^. All the 6 fractions (Fr_1_-Fr_6_) were analyzed by LC-MS/MS and employed for neuroprotective evaluation of Maca.

Maca samples (fine powder) from different places (Table S11) were weighed 1 g and dissolved in 5 mL methanol. After ultrasonic extraction for 30 min, the samples were centrifuged at 12000 g for 10 min. Then the supernatant of each sample was transferred to a clean test tube.

#### Preparation of standard solutions

The 15 reference standards were dissolved in HPLC grade methanol to achieve a stock solution with concentration of 1.0 mg.mL^−1^ for each compound respectively, then added corresponding volume according to their proportions in the sample and mixed. An internal standard stock solution was also prepared in a concentration of 0.92 mg.mL^−1^ for IS_1_ and 2.49 mg.mL^−1^ for IS_2_. The standard stock solutions were kept at 4 °C before analysis.

### Analytical conditions

In qualitative analysis, the assay was performed using an ultimate 3000 hyperbaric liquid chromatography system coupled to a LTQ Orbitrap mass spectrometer *via* an ESI interface. The chromatography system consisted of an autosampler, a diode array detector, a column compartment and two pumps. Xcalibur, Metworks and Mass Frontier 7.0 software package were used for data collection and data analysis.

Liquid chromatographic separations of the analytes were performed by a Thermo AcclaimTM120 C18 column (250 mm × 2.1 mm, 3 μm). The mobile phase consisted of 0.1% formic acid in water (solvent A) and acetonitrile (solvent B). The samples were eluted with the following linear gradient: 1% B at 0–5 min, 1–30% B at 5–20 min, 30–50% B at 20–30 min, 50–70% B at 30–40 min, 70–95% B at 40–60 min, 95% B at 60–80 min. The flow rate was 0.3 mL min^−1^. The injection volume was 5 μL. The temperature-controlled column oven was set at 30 °C and the sampler was set at 4 °C.

The ESI source parameters were as follows: Both positive and negative ionization modes were used in the analysis. For positive mode, the capillary temperature was 350 °C, shealth gas (N_2_) flow rate was 40 psi and aux gas flow rate was 10 psi, ion spray voltage was set at 3.5 kv. While for negative mode, the capillary temperature was 350 °C, shealth gas (N_2_) flow rate was 35 psi and aux gas flow rate was 10 psi, ion spray voltage was set at −3.2 kv. In the FT cell, full MS scans were acquired in the range of *m*/*z* 50–1500 with a mass resolution of 30,000. The MS/MS and MS^3^ experiments were set as data dependent scan.

In the quantitative analysis, an Agilent 6490 A triple quadrupole LC-MS system (Agilent Corporation, MA, USA) equipped with G1311A quaternary pump, G1322A vacuum degasser, G1329A autosampler and G1316A therm was employed.

Chromatography was performed on a Thermo Hypersil Gold-C18 column (2.1 mm × 50 mm, 1.9 μm) with the column temperature at 30 °C. Mobile phase consisted of water containing 0.2% formic acid (A) and acetonitrile (B) and pumped at a flow rate of 0.3 mL.min^−1^. A gradient program was used as follows: 1% B at 0–1 min, 1–5% B at 1–2 min, 5–10% B at 2–4 min, 10–50% B at 4–7 min, 50–90% B at 7–10 min, 90–100% B at 10–13 min, 100% B at 13–15 min. The injection volume was 2 μL.

Analytes were quantitated by monitoring the precursor-product combination in the DMRM mode using ion polarity switching mode. To ensure the desired abundance of each compound, the CE values and other parameters were optimized and illustrated as follows: cycle time, 300 ms; For positive mode, capillary voltage, 3 kv, nozzle voltage, 1.5 kv, Delta EMV(+), 200 v. For negative mode, capillary voltage, −2 kv, nozzle voltage, −1 kv, Delta EMV(−), 200 v. The optimized mass transition ion pairs (*m*/*z*) for analytes and the detection of the conditions of the compounds were shown in Table 4.

#### Neuroprotective effect screening of fractions and compounds in Maca

Stock solutions of MPTP (10 mg.mL^−1^) were made by adding water directly to the bottle. MPTP was diluted in Holtfreter’s solution to achieve final concentration of 200 μM.

The samples for neuroprotective effect of Maca in zebrafish including total extract, aforementioned 6 fractions and two pure compounds (A_5_, A_54_) were dissolved in DMSO to get stock solution according to their toxic limit. Nomifensine, a dopamine transporter (DAT) inhibitor as positive control, was dissolved in DMSO to get the concentration of 3 μM. The content of DMSO in solutions was not more than 0.5%.

The neuroprotective effect assessment was performed on zebrafish model treated with 200 μM MPTP. Wild-type zebrafish embryos (1 days post fertilization (dpf)) were treated with pure compounds or fractionsin zebrafish model for 2 days. After treatment, *anti-tyrosine hydroxylase (TH) whole-mount immunostaining* was performed using literature reported method^57^,^58^. Zebrafish were fixed in 4% paraformaldehyde in PBS for 5 h, rinsed, and stored at −20 °C in 100% ethanol. Briefly, fixed samples were blocked (2% lamb serum and 0.1% BSA in PBST) for 1 h at room temperature. A mouse monoclonal anti-TH antibody (1:200 diluted in blocking buffer, MAB318, Millipore) was used as the primary antibody and incubated with the sample overnight at 4 °C. The next day, samples were washed 6 times with PBST (30 min each wash), followed by incubation with goat anti-mouse antibody (1: 500 diluted in blocking buffer, Alexa Fluor^®^, USA) as secondary antibody for 1 h at room temperature in the dark. After staining, zebrafish were flat-mounted with 3.5% methylcellulose and photographed.

The experimental results of each fraction and compound were obtained from the three zebrafish statistics. Every zebrafish was taken a picture and the gray scale was calculated by IMAGE J software. As a result, representative pictures of dopaminergic neurons in the zebrafish brain from different treatment groups were selected. TH^+^ neurons in the diencephalic area of the zebrafish brain were considered as DA neurons.

For quantification of neuronal area, the periphery of each cluster was outlined by manually tracing the edge. The area (Am^2^) of each enclosed region was measured, and the subsections were summed to give the total cluster area. Origin software (MicroCal Software, Inc.) was used to generate graphs. Statistical significance was obtained by performing one-way ANOVA test using SPSS software.

Quantitative analysis of TH^+^ neurons was examined in each treatment groups. Values were expressed as a percentage of the control. #p < 0.05 vs. control; *p < 0.05 and **p < 0.01 vs. MPTP group.

#### AChE and BuChE inhibition assay

A modified Elman’s test was performed to investigate the AChE and BuChE inhibitory potency of fractions and compounds^59^. The data was measured in Microtiter plates 96 wells (Sterilin Art, No.611F96) and recorded on 1420 multilabel counter (Perkin Elmer, Wallac Victor^2^). The solutions of enzyme (at 2.43 units.mL^−1^ concentration) were prepared in Trizma buffer (7.09 mg.mL^−1^, pH 8). The solutions of reference (eserin), pure compounds and fractions (20 μL each, at 0.025 to 1000 μg.mL^−1^ concentration) in DMSO and BuChE/AChE (40 μL) were added to buffer (190 μL) and incubated at 25 °C for 5 min. DTNB (20 μL) and acetylthiocholine iodide (ATC) (20 μL) were added to enzyme-inhibitor mixture to investigate the reaction. The production of yellow anion was determined for 10 min at 405 nm. By using same methodology, similar solution of enzyme without the inhibitor was processed, which acted as a control. The blank measurement consisted of substrate (20 μL), DTNB (20 μL), DMSO (20 μL) and buffer (230 μL). The experiment was done triplicate. The percentage inhibition was calculated using the following equation:

Inhibition% = {(Positive control_A_-Blank_A_)-(Sample_A_-Blank_A_)}/(Positive control_A_-Blank_A_) = absorption

Results were expressed as mean ± SD. Differences among the groups were subjected to a one-way ANOVA (analysis of variance) followed by Duncan’s multiple range. Statistical significance was accepted when a *p*-value was less than 0.05.

### Ethics statement

We confirm that all methods were carried out in accordance with relevant guidelines and regulations.

We confirm that all experimental protocols were approved by Medicine Ethics Committee in Institute of Chinese Materia Medica, China Academy of Chinese Medical Sciences.

## Additional Information

**How to cite this article**: Zhou, Y. *et al*. Chemical profiling analysis of Maca using UHPLC-ESI-Orbitrap MS coupled with UHPLC-ESI-QqQ MS and the neuroprotective study on its active ingredients. *Sci. Rep.* **7**, 44660; doi: 10.1038/srep44660 (2017).

**Publisher's note:** Springer Nature remains neutral with regard to jurisdictional claims in published maps and institutional affiliations.

## Supplementary Material

Supplementary File

## Figures and Tables

**Table 1:** Linear equation, linear range, correlation coefficient and detection limits of 15 constituents.

Compounds	Regression equations	Linear ranges (μg.mL^−1^)	Correlation coefficients/r	Detection Limits (ng.mL^−1^)	Quantification Limits (ng.mL^−1^)
G_5_	y = 0.98x + 0.40	13.14~2628.87	0.9952	26.29	262.89
G_3_	y = 5.43x + 7.39e^−5^	0.93~186.43	0.9997	18.64	186.43
G_8_	y = 5.63x + 2.07e^−3^	0.24~23.80	0.9998	118.99	237.97
A_52_	y = 0.21x + 0.10	2.39~478.52	0.9990	4.79	4.79
A_8_	y = 8.67x + 0.40	0.18~35.14	0.9964	0.35	0.35
A_54_	y = 1.14x + 5.67	1.92~384.88	0.9951	3.85	3.85
O_6_	y = 1.02x + 7.15e^−3^	5.65e^−2^~11.30	0.9989	0.11	0.11
A_66_	y = 1.99x + 0.16	0.30~60.14	0.9956	0.60	0.60
A_67_	y = 0.62x + 2.83e^−2^	0.22~43.64	0.9960	0.44	4.36
A_5_	y = 1.44x + 0.24	0.45~90.38	0.9954	0.90	0.90
A_4_	y = 3.67x + 6.86e^−2^	0.13~26.29	0.9985	0.26	0.26
C_3_	y = 0.83x + 1.28e^−2^	0.72~1433.68	0.9974	143.37	286.74
C_5_	y = 2.32x + 1.78e^−3^	0.82~8.25	0.9957	164.95	824.74
A_121_	y = 3.42x + 1.68e^−2^	2.46e^−2^~4.91	0.9981	0.05	0.05
O_9_	y = 1.38x + 5.48e^−2^	0.18~36.08	0.9985	0.36	0.36

**Table 2:** The contents of 15 compounds in 17 samples of Maca (μg.g^−1^, n = 3).

Compounds	1	2	3	4	5	6	7	8	9	10	11	12	13	14	15	16	17	Leaves of Maca
G_5_	1.35e^4^	7.29e^4^	1.35e^4^	4.43e^4^	1.19e^4^	6.77e^4^	3.72e^4^	6.87e^4^	4.90e^4^	4.56e^4^	1.05e^4^	3.35e^4^	4.15e^4^	6.64e^4^	4.66e^4^	2.37e^4^	7.46e^4^	1.67e^4^
G_3_	3.21e^3^	4.30e^3^	2.59e^3^	1.61e^3^	6.87e^2^	2.50e^3^	1.44e^3^	3.16e^3^	1.76e^3^	2.02e^3^	4.24e^2^	1.20e^3^	1.80e^3^	3.17e^3^	1.37e^3^	9.30e^2^	4.15e^3^	6.28e^2^
G_8_	50.39	646.70	612.04	282.16	104.27	455.12	371.59	576.75	496.56	400.90	90.58	309.11	368.97	1.13 e^3^	418.06	433.26	1.04 e^3^	220.77
A_52_	955.27	1.00 e^3^	1.01 e^3^	317.23	487.52	864.58	920.38	1.92 e^3^	1.01 e^3^	1.58 e^3^	483.78	830.26	1.33 e^3^	1.59 e^3^	366.69	2.21 e^3^	778.60	257.88
A_8_	2.26	1.52	2.48	—	0.81	—	12.07	0	0.95	1.03	1.21	1.39	1.01	—	0.88	1.42	1.54	1.47
A_54_	918.95	397.09	518.27	234.68	232.52	661.36	413.40	369.04	337.46	496.81	352.89	287.28	482.69	591.76	167.21	685.48	349.95	96.85
O_6_	0.32	—	0.32	—	0.28	—	—	0.40	—	—	—	—	—	0.29	—	0.89	—	—
A_66_	82.83	10.27	43.34	3.30	32.87	50.48	7.92	8.42	8.59	28.07	40.67	10.61	20.72	10.15	1.47	31.80	5.77	—
A_67_	12.81	2.56	8.41	—	9.48	11.15	2.25	2.29	2.71	9.01	12.76	3.40	7.08	2.29	—	12.04	1.65	—
A_5_	40.24	11.03	29.63	3.50	21.47	19.62	18.88	24.98	19.81	20.37	8.64	9.96	37.05	53.40	3.39	44.95	5.39	14.96
A_4_	25.13	27.59	16.06	0.10	8.00	52.99	4.40	12.89	5.51	3.37	10.28	9.11	2.30	6.71	3.66	3.42	14.19	0.69
C_3_	23.30	6.62	35.38	—	20.74	16.47	10.46	9.16	7.01	11.59	7.85	6.38	6.37	29.73	5.68	20.05	13.50	18.39
C_5_	78.85	35.08	143.64	21.92	124.84	187.08	67.09	90.60	43.11	144.54	72.13	66.90	60.85	101.40	86.34	206.56	237.07	595.44
A_121_	0.19	0.13	0.51	—	1.60	1.01	—	0.32	—	0.22	0.58	—	0.14	0.43	—	0.26	0.25	0.13
O_9_	3.51	2.60	2.42	1.16	4.12	2.65	2.61	3.76	2.45	2.54	2.80	2.13	3.03	3.86	0.99	2.05	2.94	—

**Table 3:** The results of Acetylcholinesterase and Butyrylcholinesterase inhibition assays of Maca samples.

NO.	Samples	Acetylcholinesterase inhibition assay IC_50_ (μg.mL^−1^)	Butyrylcholinesterase inhibition assay IC_50_ (μg.mL^−1^)
0	Standard (eserin)	0.46	0.58
1	Total methanol extract	100.43	42.25*
2	Fr_1_	466.83	318.42
3	Fr_2_	663	281.68
4	Fr_3_	169.83	233.16
5	Fr_4_	50.78*	45.11*
6	Fr_5_	5.37**	5.41**
7	Fr_6_	15.77**	23.39**
8	A_54_	14.23**	17.54**

*p < 0.05 and **p < 0.01.

**Table 4:** Parameters of UHPLC-ESI-QqQ MS analysis for 15 constituents.

Compounds	Precursor Ion → Product Ion/(*m*/*z*)	Collision Energy/V
G_5_	407.8 → 74.9; 96.9*	37; 24
G_3_	437.9 → 74.9; 97.0*	34; 34
G_8_	423.8 → 75.0; 96.9*	30; 30
A_52_	376.2 → 121.0*; 238.9	24; 18
A_8_	212.0 → 91.0*; 105.0	30; 12
A_54_	346.2 → 91.0*; 239.1	24; 24
O_6_	339.0 → 121.0*; 297.1	34; 18
A_66_	216.0 → 91.0; 198.0*	37; 6
A_67_	228.0 → 94.0; 200.0	21; 15
A_5_	150.0 → 65.0; 91.0*	46; 18
A_4_	136.0 → 65.0; 91.0*	40; 21
C_3_	121.9 → 50.8; 78.0*	30; 6
C_5_	116.9 → 73.0*; 78.9	6; 6
A_121_	211.0 → 169.0; 193.0*	30; 30
O_9_	279.1 → 149.0*; 205.1	15; 3

*Quantitative ion.

## References

[b1] Leon J. The “Maca” (*Lepidium meyenii*), A little known food plant of Peru. Econ Bot 122–127 (1963).

[b2] Gustavo F. G. . Maca (*Lepidium meyenii* Walp), A review of its biological properties. Rev Peru Med Exp Salud Publica 31, 100–105 (2014). 24718534

[b3] Xiao W. . Recent Advances in study of Peruvian Lepidium meyenii (maca). World Science and Technology/Modernization of Traditional Chinese Medicine and Materia Medica 9, 102–106 (2007).

[b4] Alejandro P. F., Diane N. & Timothy J. M. Neuroprotective effects of *Lepidium meyenii* (Maca). Annals of the New York Academy of Sciences 1199, 77–85 (2010). 20633111 10.1111/j.1749-6632.2009.05174.x

[b5] Almukadi H. . The macamide N-3-methoxybenzyl-linoleamide is a time-dependent fatty acid amide hydrolase (FAAH) inhibitor. Mol Neurobiol 48, 333–339 (2013). 23853040 10.1007/s12035-013-8499-2

[b6] Wu H. . Macamides and their synthetic analogs: Evaluation of *in vitro* FAAH inhibition. Bioorg Med Chem 21, 5188–5197 (2013). 23891163 10.1016/j.bmc.2013.06.034

[b7] Robio J. . Aqueous and hydroalcoholic extracts of Black Maca (*Lepidium meyenii*) improve scopolamine-induced memory impairment in mice. Food Chem Toxicol 45, 1882–1890 (2007). 17543435 10.1016/j.fct.2007.04.002

[b8] Rubio J. . Effect of three different cultivars of *Lepidium meyenii* (Maca) on learning and depression in ovariectomized mice. BMC Complem Altern M 6, 23 (2006). 10.1186/1472-6882-6-23 PMC1534053 16796734

[b9] Rubio J. . Dose-response effect of black maca (*Lepidium meyenii*) in mice with memory impairment induced by ethanol. Toxicol Mech Method 21, 628–634 (2011). 10.3109/15376516.2011.583294 21780878

[b10] Rubio J., Qiong W. & Liu X. M. Aqueous Extract of Black Maca (*Lepidium meyenii*) on memory impairment induced by ovariectomy in Mice. Evid-based Compl Alt 1–7 (2011). 10.1093/ecam/nen063 PMC3096456 18955369

[b11] Megan M. M. . Analysis of macamides in samples of Maca (*Lepidium meyenii*) by HPLC-UV-MS/MS. Phytochem Anal 16, 463–469 (2005). 16315492 10.1002/pca.871

[b12] Haidu Z. . Identification of endocannabinoid system-modulating N-alkylamides from Heliopsis helianthoides var. scabra and *Lepidium meyenii*. J Nat Prod. 77, 663–1669 (2014). 24972328 10.1021/np500292g

[b13] Nie D. S. . The study of *Lepidium meyenii* (Maca) on sexual function and related health benefits. J of Human Sexual 22, 10–12 (2013).

[b14] Bai N. . Study on the composition and pharmacological effects of Cruciferousplants. J Chinese Med Mat 34, 1465–1468 (2011).

[b15] Brian P. W. & Randall T. P. Zebrafish models of cerebrovascular disease. J Cerebr Blood F Met, 34, 571–577 (2014). 10.1038/jcbfm.2014.27 PMC3982096 24517974

[b16] Edor K., Edna B., Nathalie C. & Pierre D. Zebrafish models for the functional genomics of neurogenetic disorders. BBA-biomembranes 1812, 335–345 (2011). 20887784 10.1016/j.bbadis.2010.09.011

[b17] Lee Y. . Improvement of pentylenetetrazol-induced learning deficits by valproic acid in the adult zebrafish. Eur J Pharmacol 643, 225–231 (2010). 20599908 10.1016/j.ejphar.2010.06.041

[b18] Lu X. L. . Protective effects of puerarin against Aß40-induced vascular dysfunction in zebrafish and human endothelial cells. Eur J Pharmacol 732, 76–85 (2014). 24690262 10.1016/j.ejphar.2014.03.030

[b19] Jeong J. Y. . Functional and developmental analysis of the blood–brain barrier in zebrafish. Brain Res Bull 75, 619–628 (2008). 18355638 10.1016/j.brainresbull.2007.10.043

[b20] Kim Y. H. . Reduced neuronal proliferation by proconvulsant drugs in the developing zebrafish brain. Neurotoxicol Teratol 32, 551–557 (2010). 20420900 10.1016/j.ntt.2010.04.054

[b21] Hung M. W. . From Omics to Drug Metabolism and High Content Screen of Natural Product in Zebrafish: A New Model for Discovery of Neuroactive Compound. Evid-Based Compl Alt 1–20 (2012). 10.1155/2012/605303 PMC3420231 22919414

[b22] Cui B. L., Lin B. L., He K. & Zheng Q. Y. Imidazole alkaloids from *Lepidium meyenii*. J Nat Prod 66, 1101–1103 (2003). 12932133 10.1021/np030031i

[b23] Muhammad I., Zhao J. P. & Dunbar C. Composition of the essential oil of *Lepidium meyenii*(Walp.). Phytochemistry 59, 105–110 (2002).11754952

[b24] Gustavo F. G. & Cynthia G. C. The Methyltetrahydro-Carbolines in Maca (*Lepidium meyenii*). Evid-Based Compl Alt 6, 315–316 (2009). 10.1093/ecam/nen041 PMC2722210 18955346

[b25] Dini I., Tenore G. C. & Dini A. Glucosinolates from Maca (*Lepidium meyenii*). Biochem Syst Ecol 30, 1087–1090 (2002).

[b26] Cataldi T. R. I., Lelario F., Orlando D. & Bufo S. A. Collison-Induced Dissociation of the A + 2 Isotope Ion Facilitates Glucosinolates Structure Elucidation by Electrospray Ionization-Tandem Mass Spectrometry with a Linear Quadrupole Ion Trap. Anal Chem 82, 5686–5696 (2010). 20521824 10.1021/ac100703w

[b27] Céline C. . Secondary metabolites in Maca as affected by hypocotyl color, cultivation history, and site. Agron J 102, 431–439 (2010).

[b28] Cui Y. M. Study on isolation, identification, efficacy and fingerprint of flavonoids from Licorice. Huanzhong University of Science and Technology (2008).

[b29] Sun Q. H. . Qualitative and quantitative analysis of the chemical constituents in Mahuang-Fuzi-Xixin decoction based on high performance liquid chromatography combined with time-of-flight mass spectrometry and triple quadrupole mass spectrometers. Biomed Chromatogr 30, 1820–1834 (2016). 27183898 10.1002/bmc.3758

[b30] Yang Y. . Chemical profiling and quantification of Chinese medicinal formula Huang-Lian-Jie-Du decoction, a systematic quality control strategy using ultra high performance liquid chromatography combined with hybrid quadrupole-orbitrap and triple quadrupole mass spectrometers. J Chromatogr A 1321, 88–99 (2013). 24231264 10.1016/j.chroma.2013.10.072

[b31] Pan Y., Zhang J., Li Hong, Wang Y. Z. & Li W. Y. Characteristic fingerprinting based on macamides for discrimination of maca (*Lepidium meyenii*) by LC/MS/MS and multivariate statistical analysis. J Sci Food Agric 96, 4475–4483 (2016). 26857797 10.1002/jsfa.7660

[b32] Eliana E. . Bioactive maca (*Lepidium meyenii*) alkamides are a result of traditional Andean postharvest drying practices. Phytochemistry 116, 138–148 (2015). 25817836 10.1016/j.phytochem.2015.02.030

[b33] Pan Y., Zhang J., Li H., Wang Y. Z. & Li W. Y. Simultaneous Analysis of Macamides in Maca (*Lipidium meyenii*) with Different Drying Process by Liquid Chromatography Tandem Mass Spectrometry. *Food Anal*. Methods 9, 1686–1695 (2016).

[b34] Zhang Z. J. . Examining the neuroprotective effects of protocatechuic acid and chrysin on *in vitro* and *in vivo* models of Parkinson disease. Free Radical Bio Med 84, 331–343 (2015). 25769424 10.1016/j.freeradbiomed.2015.02.030

[b35] Choi E. H. . Supplementation of standardized lipid-soluble extract from maca (*Lepidium meyenii*) increases swimming endurance capacity in rats. J Funct Foods 4, 568–573 (2012).

[b36] Pino-Figueroa A., Vu H., Kelley C. J. & Maher T. J. Mechanism of action of Lepidium meyenii (Maca): an explanation for its neuroprotective activity. Am J Neuroprotec Neuroregen 3, 87–92 (2011).

[b37] Enid T. M. . Neuroprotection of MPTP-induced toxicity in zebrafish dopaminergic neurons. Mol Brain Res 141, 128–137 (2005). 16209898 10.1016/j.molbrainres.2005.08.014

[b38] Cuevas E. . AcetylL-carnitine protects motor neurons and Rohon-Beard sensoryneurons against ketamine-induced neurotoxicity in zebrafish embryos. Neurotoxicol Teratol 39, 69–6 (2013). 23896048 10.1016/j.ntt.2013.07.005 PMC5467697

[b39] Rosemberg D. B. . Behavioral effects of taurine pretreatment in zebrafish acutely exposed to ethanol. Neuropharmacol 63, 613–623 (2012). 10.1016/j.neuropharm.2012.05.009 22634362

[b40] Rosemberg D. B. . Taurine Prevents Enhancement of Acetylcholinesterase Activity Induced by Acute Ethanol Exposure and Decreases the Level of Markers of Oxidative Stress in Zebrafish Brain. Neuroscience 171, 683–692 (2010). 20884336 10.1016/j.neuroscience.2010.09.030

[b41] Edor K., Edna B., Nathalie C. & Pierre D. Zebrafish models for the functional genomics of neurogenetic disorders. Biochim Biophys Acta 1812, 335–345 (2011). 20887784 10.1016/j.bbadis.2010.09.011

[b42] Bretaud S., Lee S. & Guo S. Sensitivity of zebrafish to environmental toxins implicated in Parkinson’s disease. Neurotoxicol Teratol 26, 857–864 (2004). 15451049 10.1016/j.ntt.2004.06.014

[b43] Liu J. C. . Necrosis inhibitor-5 (NecroX-5), attenuates MPTP-induced motor deficits in a zebrafish model of Parkinson’s disease. Genes Genom 37, 1073–1079 (2015).

[b44] Pan-Montojo F. & Funk R. H. Implications of Parkinson’s Disease Pathophysiology for the Development of Cell Replacement Strategies and Drug Discovery in Neurodegenerative Disease. CNS Neurol Disord-DR 11, 907–920 (2012). 10.2174/1871527311201070907 23131153

[b45] Ming-Wai H. . From Omics to Drug Metabolism and High Content Screen of Natural Product in Zebrafish: A New Model for Discovery of Neuroactive Compound. Evidence-Based Complementary and Alternative Medicine 1–20 (2012). 10.1155/2012/605303 PMC3420231 22919414

[b46] Hiliario W. F. . Cholinergic and Dopaminergic Alterations in Nigrostriatal Neurons Are Involved in Environmental Enrichment Motor Protection in a Mouse Model of Parkinson’s Disease. J Mol Neurosic 60, 453–464 (2016). 10.1007/s12031-016-0831-7 27660217

[b47] Degroot A. & Treit D. Septal GABAergic and hippocampal cholinergic systems interact in the modulation of anxiety. Neuroscience 117, 493–501 (2003). 12614689 10.1016/s0306-4522(02)00651-6

[b48] Nardone R., Höller Y., Thomschewski A., Kunz A. B., Lochner P. & Golaszewski S. Dopamine differently modulates central cholinergic circuits in patients with Alzheimer disease and CADASIL. J Neural Transm 121, 1313–1320 (2014). 24677024 10.1007/s00702-014-1195-1

[b49] Martorana A. . Dopamine Modulates Cholinergic Cortical Excitability in Alzheimer’s Disease Patients. Neuropsychopharm 34, 2323–2328 (2009). 10.1038/npp.2009.60 19516251

[b50] Wu H. . Macamides and their synthetic analogs: Evaluation of *in vitro* FAAH inhibition. Bioorg Med Chem 21, 5188–5197 (2013). 23891163 10.1016/j.bmc.2013.06.034

[b51] Stelt M. V. & Marzo V. D. The endocannabinoid system in the basal ganglia and in the mesolimbic reward system: Implications for neurological and psychiatric disorders. Eur. J. Pharmacol 480, 133 (2003). 14623357 10.1016/j.ejphar.2003.08.101

[b52] Mazzola J. C. . Fatty acid amide hydrolase (FAAH) inhibition enhances memory acquisition through activation of PPAR-alpha nuclear receptors. Learn Mem. 16, 332 (2009). 19403796 10.1101/lm.1145209 PMC2683005

[b53] Montanari S. . Fatty Acid Amide Hydrolase (FAAH), Acetylcholinesterase (AChE), and Butyrylcholinesterase (BuChE): Networked Targets for the Development of Carbamates as Potential Anti-Alzheimer’s Disease Agent. J med chem 59, 6387–6406 (2016). 27309570 10.1021/acs.jmedchem.6b00609

[b54] Pan L. . Design, synthesis and evaluation of isaindigotone derivatives as acetylcholinesterase and butyrylcholinesterase inhibitors. Bioorg & Med Chem Lett 18, 3790–3793 (2008). 18524585 10.1016/j.bmcl.2008.05.039

[b55] Zhou Y. Y. . Recent researching progress of *Lepidium meyenii* (Maca). China Journal of Chinese Materia Medica 40, 4521–4530 (2015). 27141658

[b56] Yang W. Z. . A strategy for efficient discovery of new natural compounds by integrating orthogonal column chromatography and liquid chromatography/mass spectrometry analysis: Its application in Panax ginseng, Panax quinquefolium and Panax notoginseng to characterize 437 potential new ginsenosides. Anal Chim Acta 739, 56–66 (2012). 22819050 10.1016/j.aca.2012.06.017

[b57] Zhang Z. J., Cheang L. C., Wang M. M. & Lee S. M. Quercetin exerts a neuroprotective effect through inhibition of the iNOS/NO system and pro-inflammation gene expression in PC12 cells and in zebrafish. Int J Mol Med 27, 195–203 (2011). 21132259 10.3892/ijmm.2010.571

[b58] Bitzur S., Kam Z. & Geiger B. Structure and distribution of N-cadherin in developing zebrafish embryos: morphogenetic effects of ectopic over-expression. Dev Dynam 201, 121–136 (1994). 10.1002/aja.1002010204 7873785

[b59] Wszelaki N., Kuciun A. & Kiss A. K. Screening of traditional European herbal medicines for acetylcholineterase and butyrylcholinesterase inhibitory activity. Acta Pharmacol 60, 119–128 (2010). 10.2478/v10007-010-0006-y 20228046

