# Supplementary material for: Chemical profiling analysis of Maca using UHPLC-ESI-Orbitrap MS coupled with UHPLC-ESI-QqQ MS and the neuroprotective study on its active ingredients
Source: Sci Rep. 2017 Mar 17;7:44660. doi: 10.1038/srep44660 (PMC5356334; doi:10.1038/srep44660)
Supplement: Supplementary File [file srep44660-s1.pdf]

Chemical profiling analysis of Maca using UHPLC-ESI-Orbitrap MS coupled with UHPLC-ESI-QqQ MS and the neuroprotective study on its active ingredients

Yanyan Zhou[1#] Peng Li[2#] Adelheid Brantner[3] Hongjie Wang[1] Xinbin Shu[4] Jian Yang[1] Nan Si[1] Lingyu Han[1] Haiyu Zhao[1*] & Baolin Bian[1*]

[1]Institute of Chinese Materia Medica, China Academy of Chinese Medical Sciences, Beijing, China

[2]Institute of Chinese Medical Sciences, University of Macau, Macau, China

[3]Institute of Pharmaceutical Sciences Pharmacognosy, University of Graz, Graz, Austria

[4]Shandong Rosemed Biopharm LTC, Yanzhou, Shandong province, China

#: These two authors contributed equally to the paper

[*]Correspondence author at: Institute of Chinese Materia Medica, China Academy of Chinese Medical Sciences

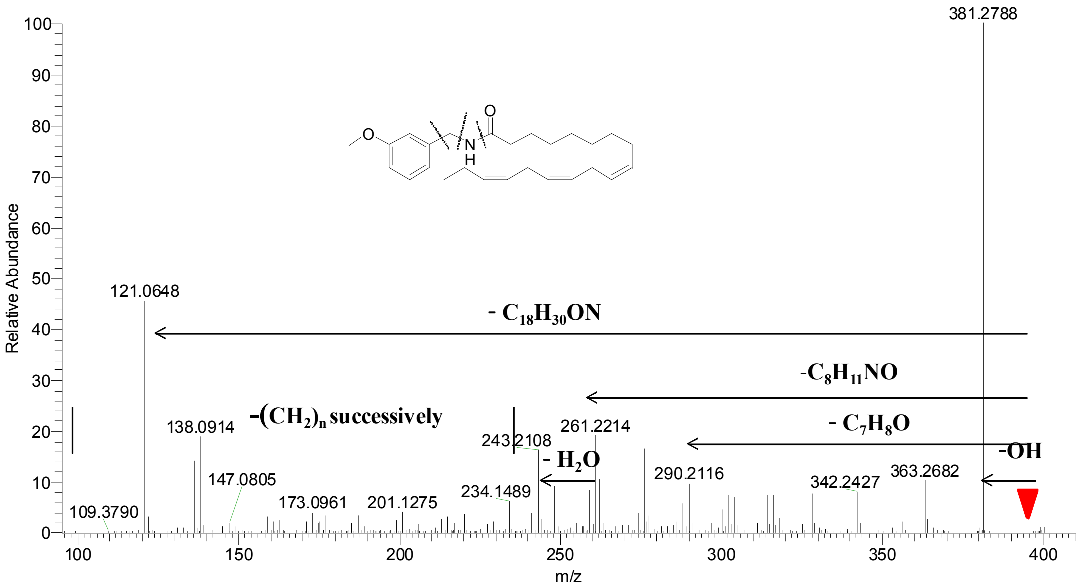

Figure S1 The MS/MS spectrum of N-(3-methoxybenzyl)-(9Z,12Z,15Z)-octadecatrienamide (No. A_{32})

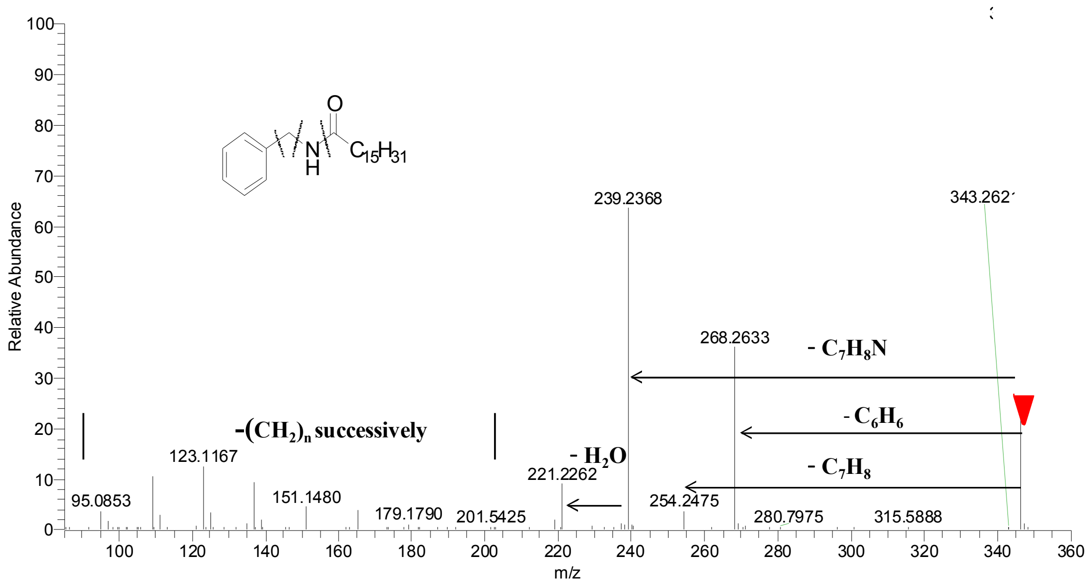

Figure S2 The MS/MS spectrum of N-benzylhexadecanamide (No. A_{54})

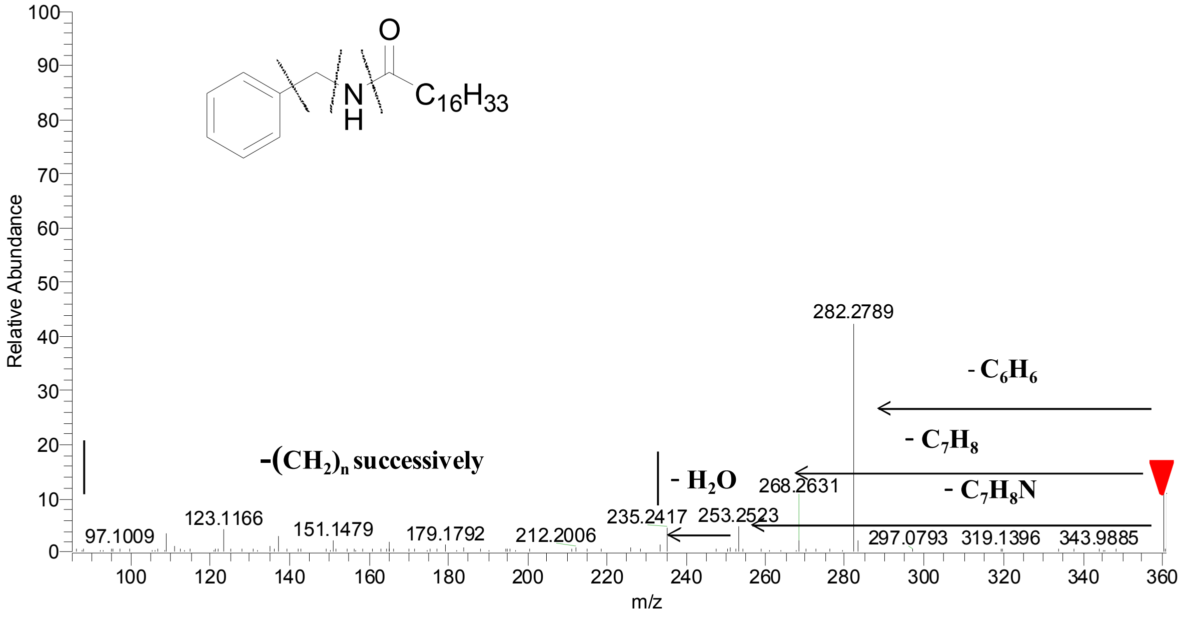

Figure S3 Proposed structures and MS/MS spectrum for N-benzylheptadecanamide (No. A_{58})

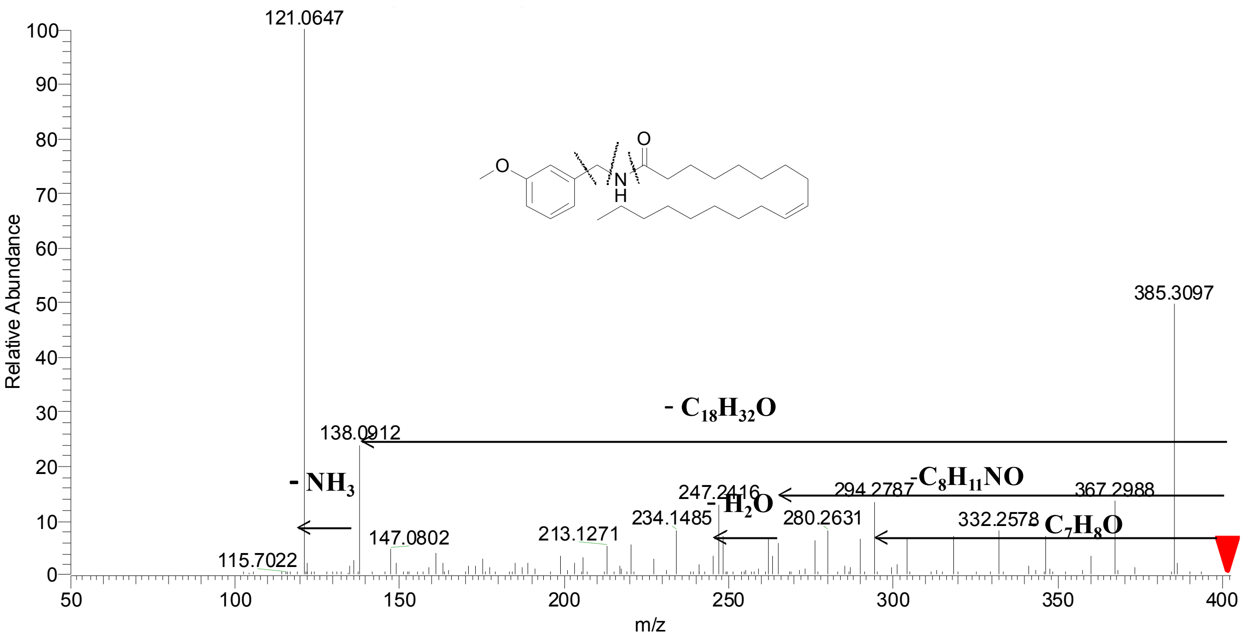

Figure S4 Proposed structures and MS/MS spectrum for N-(3-methoxybenzyl)-(9Z)-octadecenamide (No.A_{55})

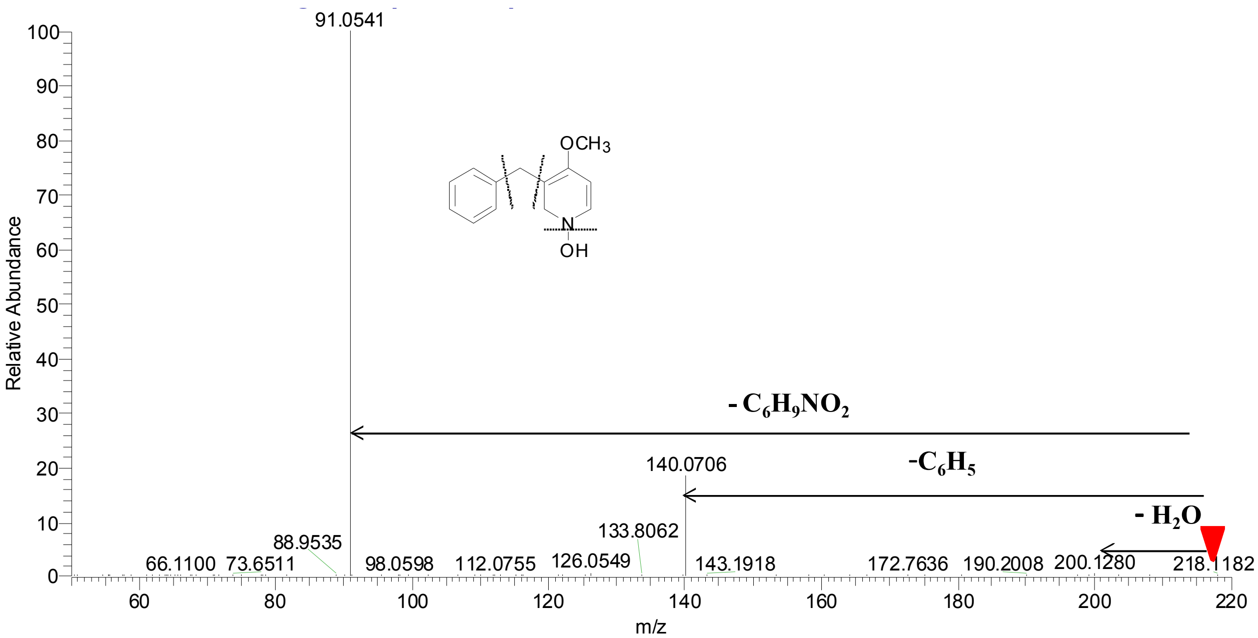

Figure S5 Proposed structures and MS/MS spectrum for 3-benzyl-1,2-dihydro-N-hydroxypyridine-4-methoxy

(No. A_{65})

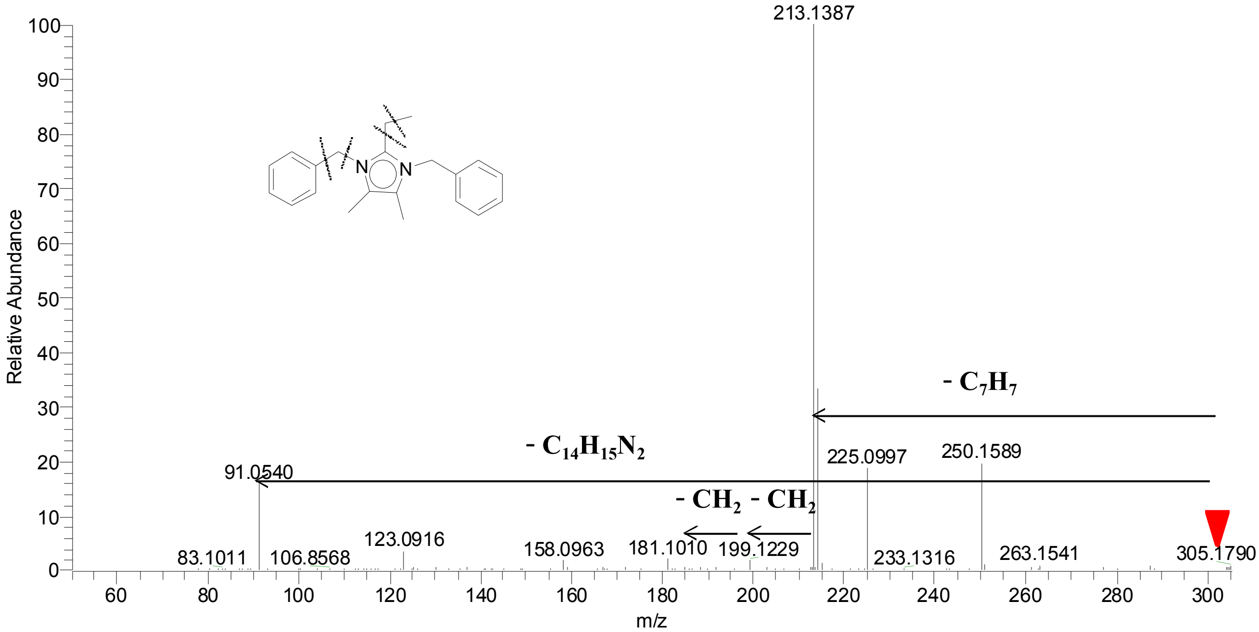

Figure S6 Proposed structures and MS/MS spectrum for 1,3-dibenzyl-2(R)-ethyl-4,5-dimethylimidazilium (No. A_{84})

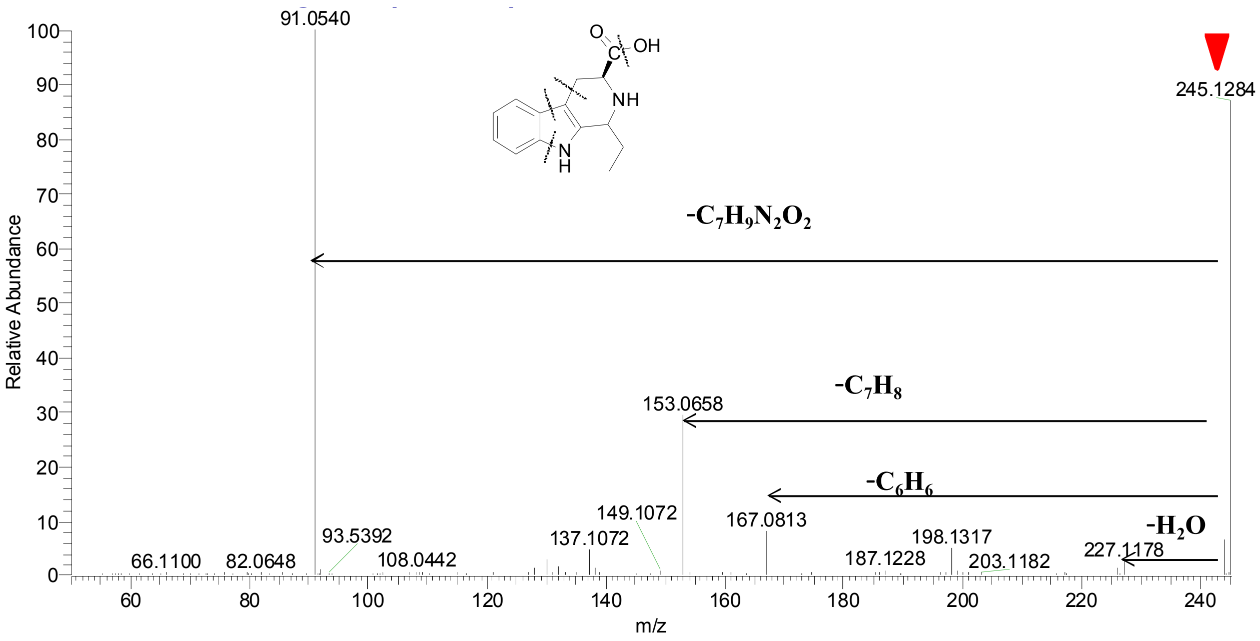

Figure S7 Proposed structures and MS/MS spectrum for (1R,3S)-1-ethyltetrahydro-β-5,6-carboline-3-carboxylic acid (No. A_{111})

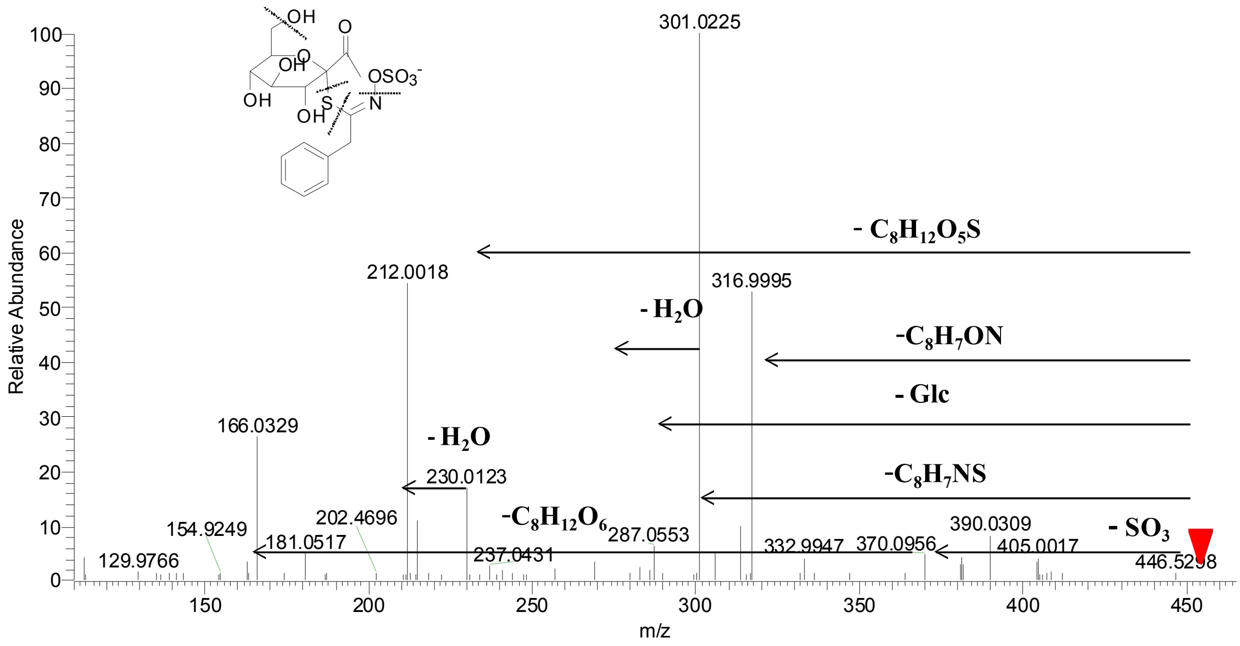

Figure S8 Proposed structures and MS/MS spectrum for acetyl-benzylglucosinolate (No. G_{11})

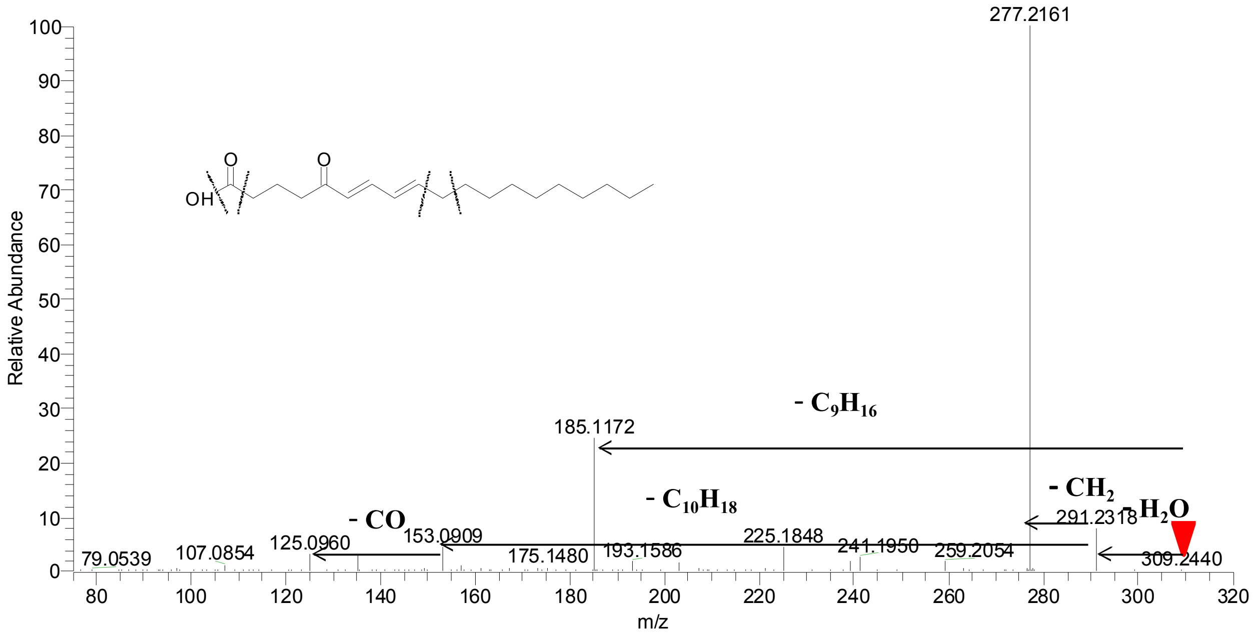

Figure S9 Proposed structures and MS/MS spectrum for 5-oxo-6E,8E-nineteencarbodienoic acid (No. M_9)

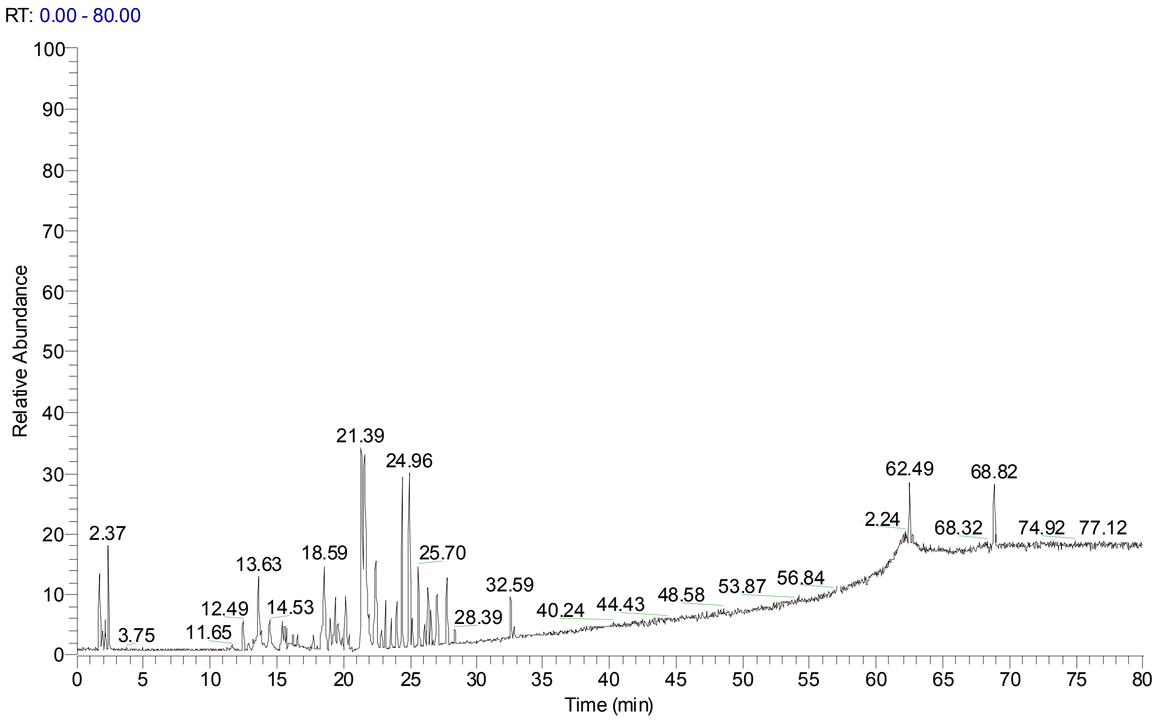

Figure S10 The LC-MS chromatogram of Fr_4

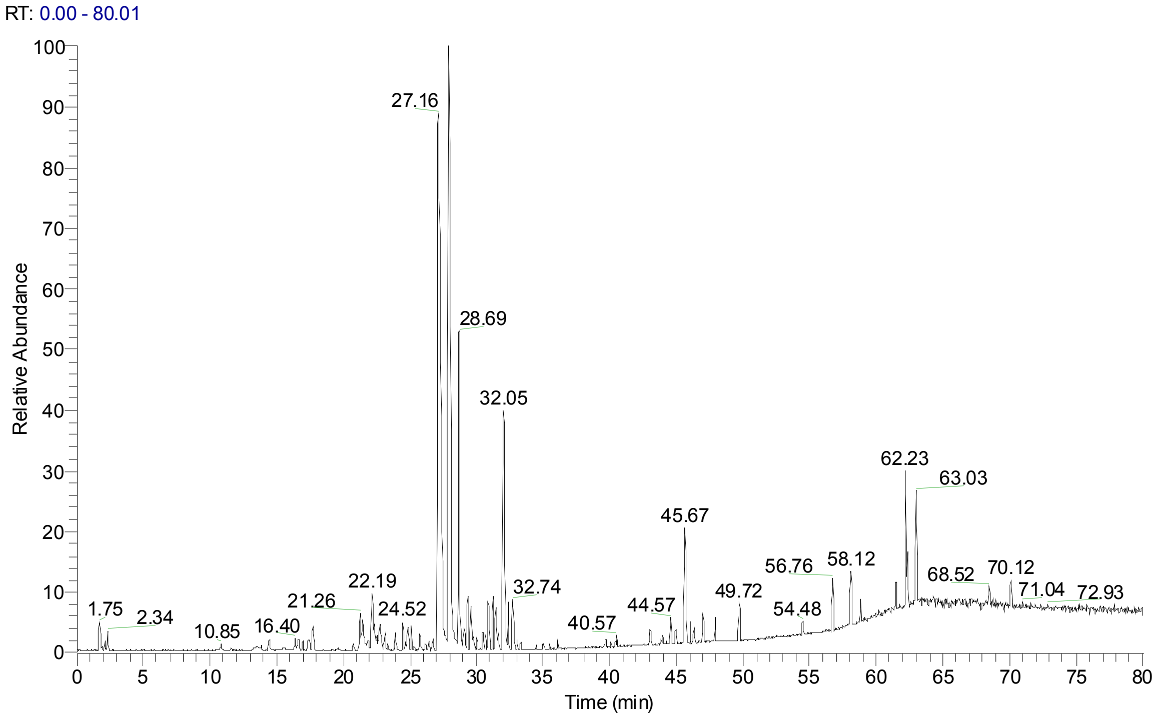

Figure S11 The LC-MS chromatogram of Fr_5

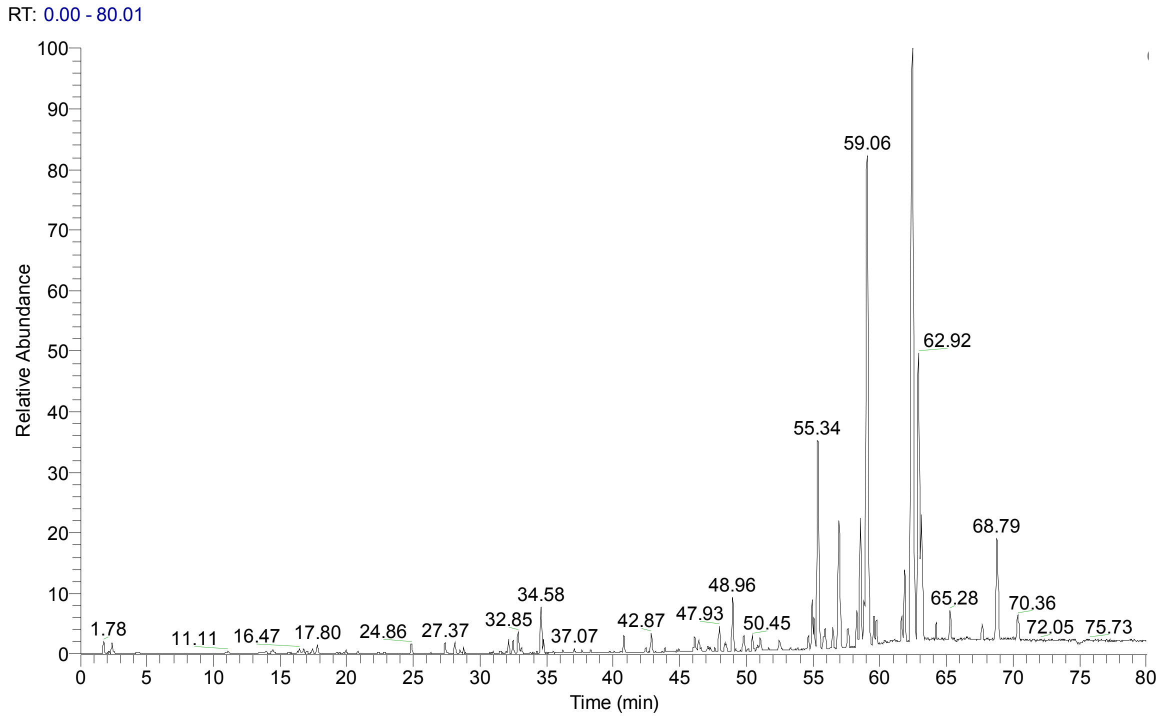

Figure S12 The LC-MS chromatogram of Fr_6

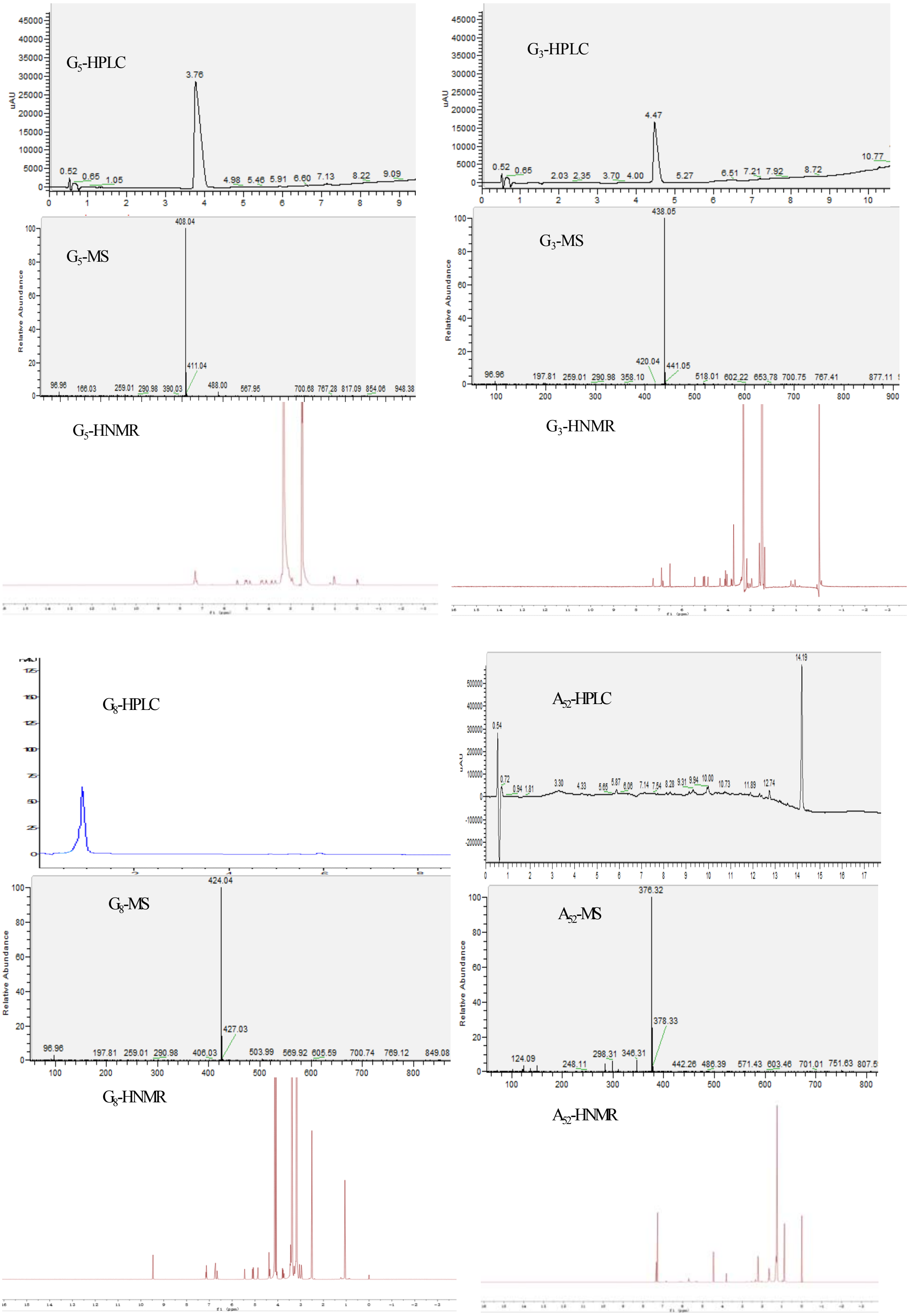
G5-HPLC
3.76
0.52 0.65 1.05 4.98 5.46 5.91 6.60 7.13 8.22 9.09
uAU
G3-HPLC
4.47
0.52 0.65 2.03 2.35 3.70 4.00 5.27 6.51 7.21 7.92 8.72 10.77
G5-MS
408.04
411.04
96.96 166.03 259.01 290.98 390.03 488.00 567.95 700.68 767.28 817.09 854.06 948.38
Relative Abundance
G3-MS
438.05
420.04 441.05
96.96 197.81 259.01 290.98 358.10 518.01 602.22 653.78 700.75 767.41 877.11
G5-HNMR
G3-HNMR
G8-HPLC
A52-HPLC
14.19
0.54 0.72 0.94 1.81 3.30 4.33 5.65 5.87 6.06 7.14 7.54 8.28 9.31 9.94 10.00 10.73 11.89 12.74
G8-MS
424.04
427.03
96.96 197.81 259.01 290.98 406.03 503.99 569.92 605.59 700.74 769.12 849.08
A52-MS
376.32
378.33
124.09 248.11 298.31 346.31 442.26 486.39 571.43 603.46 701.01 751.63
G8-HNMR
A52-HNMR

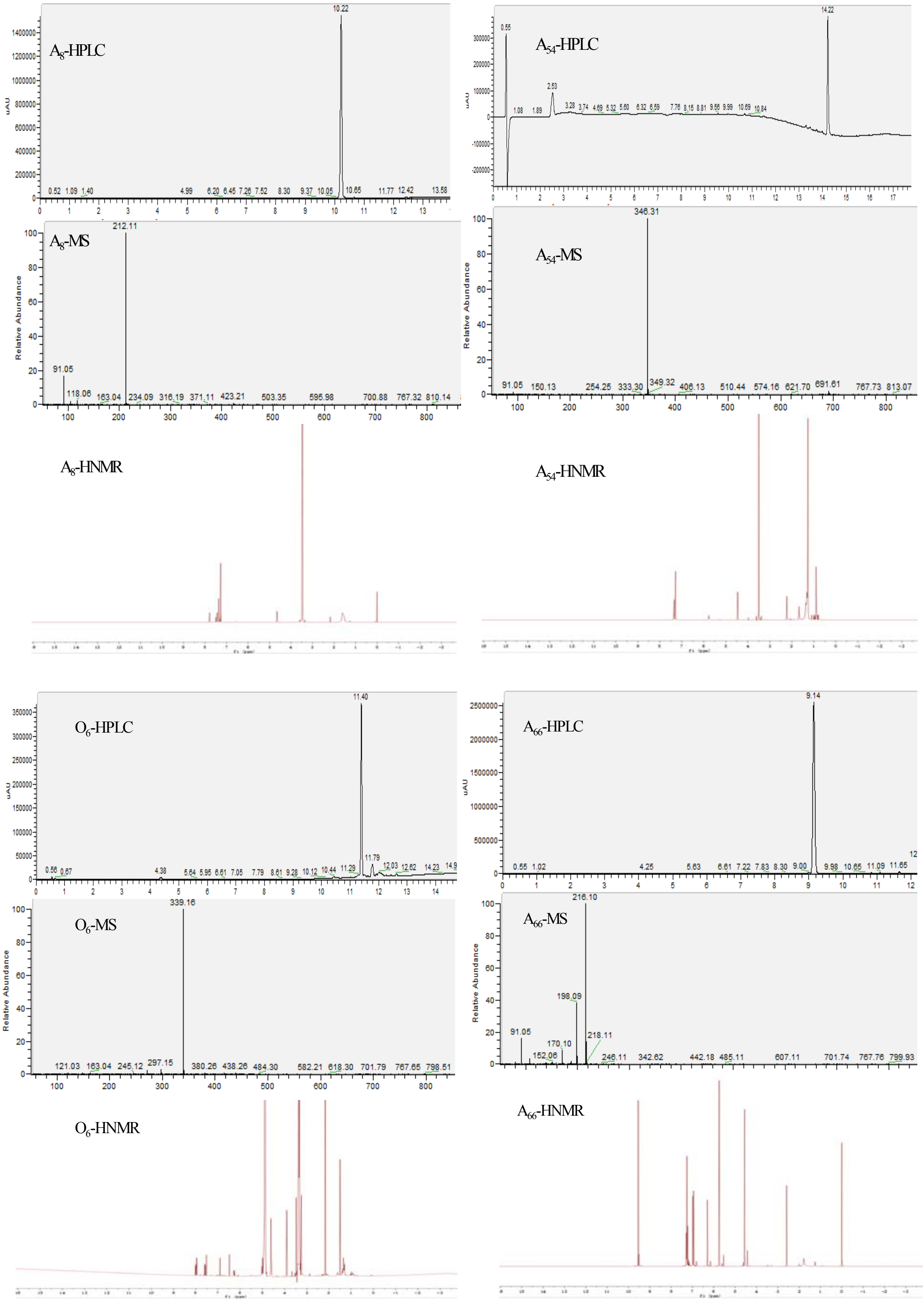

A8-HPLC
A8-MS
A8-HNMR
A54-HPLC
A54-MS
A54-HNMR
O6-HPLC
O6-MS
O6-HNMR
A66-HPLC
A66-MS
A66-HNMR

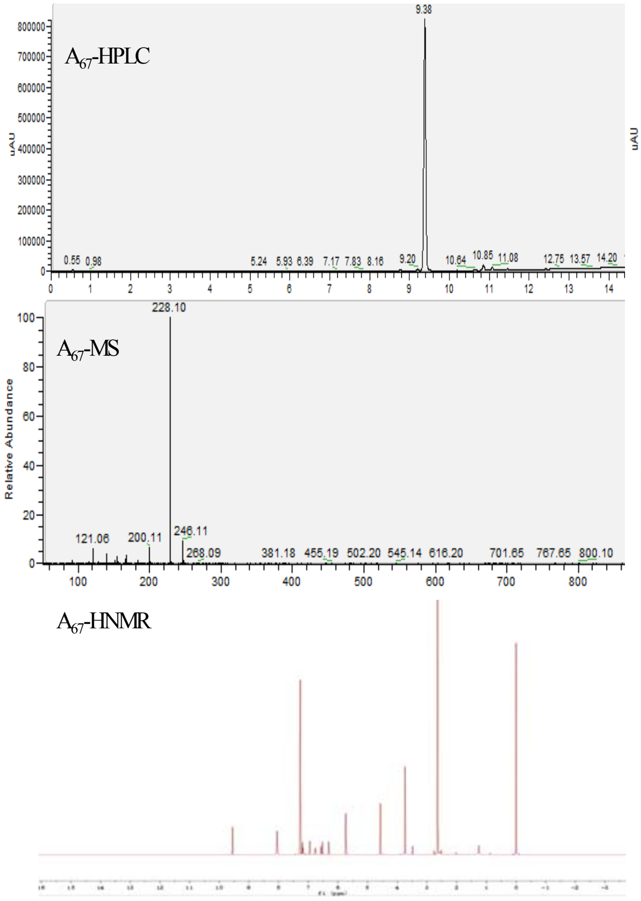
A67-HPLC
9.38
0.55 0.98 5.24 5.93 6.39 7.17 7.83 8.16 9.20 10.64 10.85 11.08 12.75 13.57 14.20
uAU
A67-MS
228.10
121.06 200.11 246.11 268.09 381.18 455.19 502.20 545.14 616.20 701.65 767.65 800.10
Relative Abundance
A67-HNMR

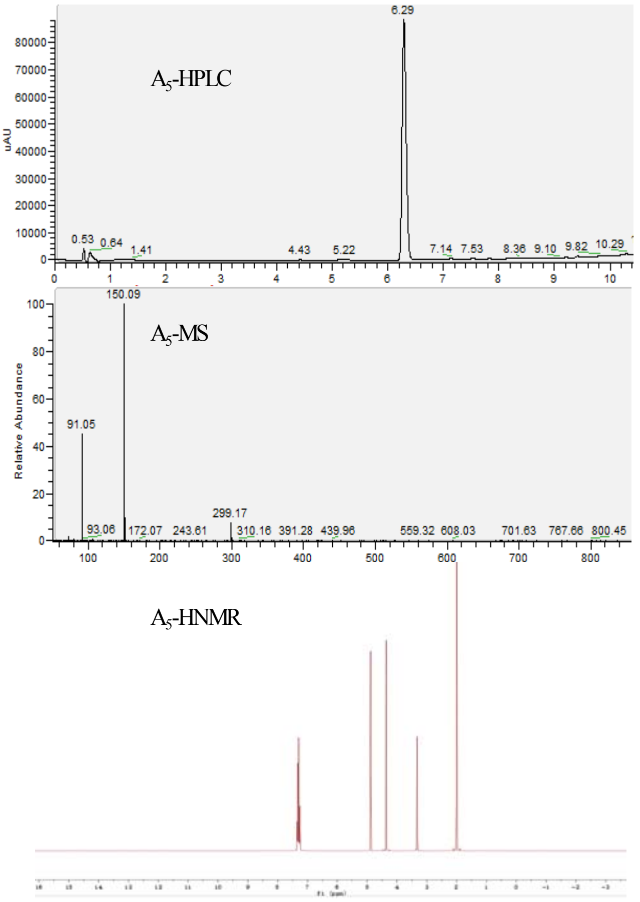
A5-HPLC
6.29
0.53 0.64 1.41 4.43 5.22 7.14 7.53 8.36 9.10 9.82 10.29
uAU
A5-MS
150.09
91.05
93.06 172.07 243.61 299.17 310.16 391.28 439.96 559.32 608.03 701.63 767.66 800.45
Relative Abundance
A5-HNMR

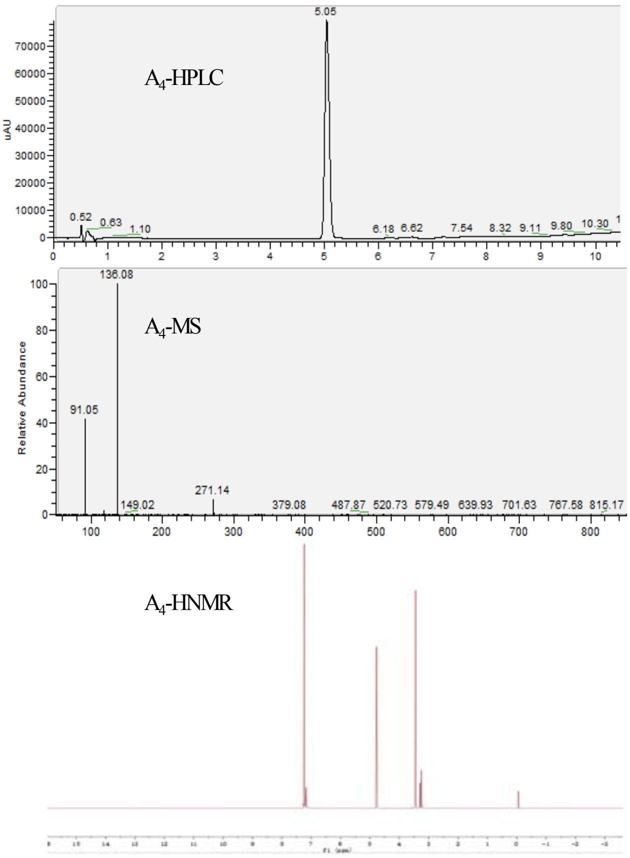
A4-HPLC
5.05
0.52 0.63 1.10 6.18 6.62 7.54 8.32 9.11 9.80 10.30
uAU
A4-MS
136.08
91.05
149.02 271.14 379.08 487.87 520.73 579.49 639.93 701.63 767.58 815.17
Relative Abundance
A4-HNMR

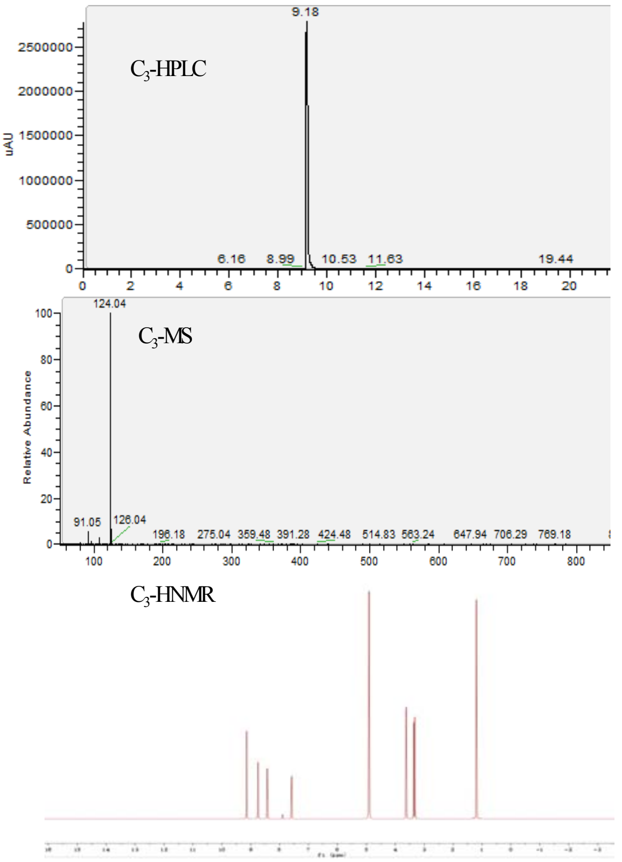
C3-HPLC
9.18
6.16 8.99 10.53 11.63 19.44
uAU
C3-MS
124.04
91.05 126.04 198.18 275.04 359.48 391.28 424.48 514.83 563.24 647.94 706.29 769.18
Relative Abundance
C3-HNMR

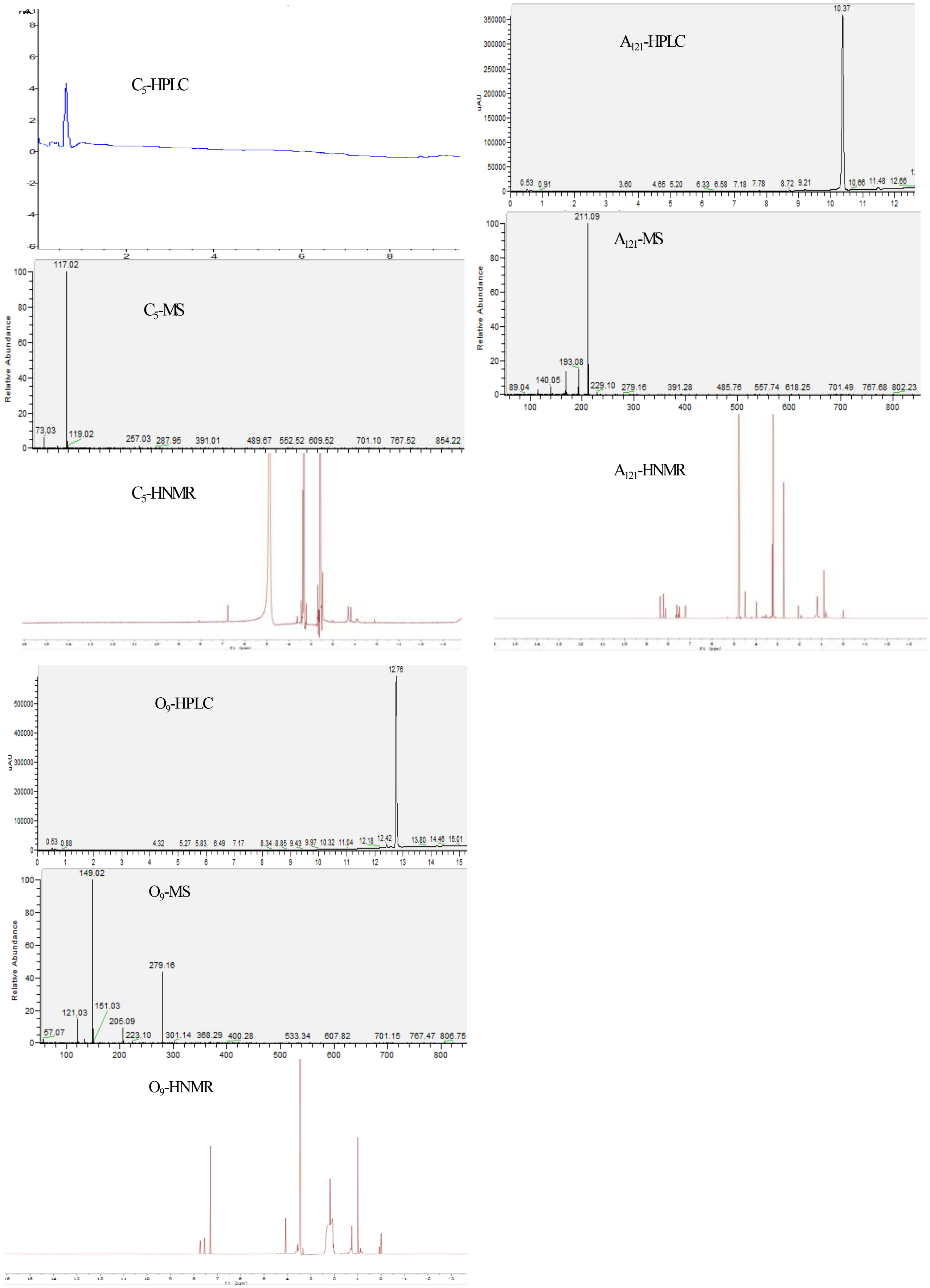

Figure S13 The HPLC, MS and HNMR spectra of 15 reference standards

Table S1 Identification of Macamides and common amide alkaloids from Maca by UHPLC-ESI-Orbitrap MS

NO	name	t_R(min)	Formula	ESI(+)/expected (*m/z*)	ESI(+)/measured (*m/z*)	Delta (ppm)	Fragamentor information
A_1^*	benzylamine	10.03	C_7H_9N	108.0807	108.0805	-2.368	**91.05405**
A_2^*	N-methyl-3-hydroxy-Benzeneacetamide	12.29	$C_9H_{11}NO_2$	166.0862	166.0859	-1.657	149.0597, 131.0491, **120.0808**, 91.0540
A_3	N-benzyl-2E,4E-fivecarbondienamide	16.35	$C_{12}H_{11}NO$	186.0913	186.0912	-0.487	168.1016, 158.0725, **126.05479**, 104.0544
A_4^*	2-phenylacetamide	17.89	C_8H_9NO	136.0756	136.0757	0.070	**119.0489**, 91.0037
A_5^*	N-acetylbenzylamine	20.04	$C_9H_{11}NO$	150.0913	150.0911	-0.151	**130.4232**, 116.0710, 91.0540
A_6	N-(3-hydroxy-benzyl)-2Z-fivecarbon acrylamide	20.08	$C_{10}H_{13}NO_2$	180.1019	180.1019	0.027	**162.0914**, 152.1071, 138.0915, 112.0757, 95.0490
A_7	N-(3-hydroxy-benzyl)-11Z,13Z,15Z,17Z-octadecatetrienamide	30.65	$C_{25}H_{35}NO_2$	382.2740	382.2735	-1.454	364.2633, 290.2114, **272.2008**, 190.1227, 173.1071
A_8^*	N-benzylbenzamide	30.71	$C_{14}H_{13}NO$	212.1069	212.1067	-0.211	134.0600, **105.0334**, 91.0540
A_9	N-benyl- Sevencarbamide	32.85	$C_{13}H_{19}NO$	206.1539	206.1539	-0.052	128.1070, 108.0807, **91.0541**, 77.9986
A_{10}	N-benzyl-2E,4E,6E-fourcarbonleukotrieneamide	34.65	$C_{14}H_{15}NO$	214.1226	214.1226	-0.190	**186.1279**, 108.0807, 91.0541
A_{11}	N-benzyl-2E,4E-sixcarbondienamide	35.58	$C_{13}H_{13}NO$	200.1069	200.1070	0.347	172.1122, 122.0601, **109.0886**, 91.0541
A_{12}	N-(3-hydroxy-benzyl)-8E,11E,13E,15E,17E-octadecapentienamide	36.21	$C_{25}H_{33}NO_2$	380.2584	380.2584	0.116	362.2480; 300.1957, **260.1646**, 131.0855, 91.0541
A_{13}	N-benzyl-octanamide	38.64	$C_{15}H_{23}ON$	234.1852	234.1852	-0.132	207.1127, 156.1382, 127.1117, 109.1010, **91.0542**
A_{14}	N-(3,4-dihydroxy-benzyl)-hexadecanamide	38.80	$C_{22}H_{41}NO_2$	364.3210	364.3204	-1.664	346.2742, 282.2065, **149.1323**, 121.1011, 91.0541
A_{15}^*	N-benzyl-(9Z,12Z,15Z)-octadecatetraenamide	43.62	$C_{25}H_{35}NO$	366.2791	366.2784	-1.997	349.2528, **259.2058**, 241.1953, 121.1012, 108.0808
A_{16}	N-(3-hydroxy-benzyl)-11E,13E,15E,17E-octadecatetrienamide	45.83	$C_{25}H_{35}NO_2$	382.2740	382.2730<br>404.2549	-2.657	364.2631, 275.2002, **260.1642**, 101.0942
A_{17}	N-benzyl-5-oxo-6E,8E-octadecadienamide[31]	47.17	$C_{25}H_{37}NO_2$	384.2897	384.2886<br>406.2704	-2.774	**366.2792**, 306.2421, 300.2323, 288.1959, 277.2162, 260.1646, 259.2057

A_{18}	N-benzyl-13-oxo-9E,11E-octadecadienamide	47.51	$C_{25}H_{37}NO_2$	384.2897	384.2891<br>406.2707	-1.525	**366.2792**, 306.2430, 286.2167, 277.2163, 259.2058
A_{19}	N-benzyl-9-oxo-12E,15Z-Octadecadienamide	48.15	$C_{25}H_{37}NO_2$	384.2897	384.2889<br>406.2707	-1.993	366.2792, 306.2433, 277.2162, **260.1646**, 259.2058
A_{20}	N-benzyl-9-oxo-12E,15E-Octadecadienamide	48.75	$C_{25}H_{37}NO_2$	384.2897	384.2885<br>406.2703	-3.304	366.2792, 306.2793, 277.2163, **260.1646**, 259.2059
A_{21}	N-butyl-14,16-dihydroxy-9E, 11E, 13E-octadecatrieneamide	48.94	$C_{22}H_{39}NO_3$	366.3002	366.2994<br>388.2811	-2.186	348.2895, **263.2368**, 245.2263, 135.1167, 121.1010
A_{22}	N-benzyl-16-hydroxy-9-oxo-3E,5E,7E-octadecatrieneamide	49.45	$C_{25}H_{35}NO_3$	398.2689	398.2685	-1.131	380.2585, 362.2491, **282.1853**, 264.1741, 246.2436
A_{23}*	N-benzyl-9-oxo-12E-octadecenamide	50.25	$C_{25}H_{39}NO_2$	386.3053	386.3045<br>408.2861	-2.216	368.2949, **351.2684**, 333.2577, 279.2320, 261.2212
A_{24}	N-benzyl-16-hydroxy-9-oxo-15E,17E-octadecatrieneamide	50.47	$C_{25}H_{35}NO_3$	398.2689	398.2780	-2.412	**380.8856**, 368.2948, 288.2323, 170.1175
A_{25}	N-eyhyl-12-(2’-4’-)-cyclohexadiene-dodeceneamide	50.76	$C_{20}H_{35}NO$	306.2791	306.2786	-1.571	289.2527, **261.2215**, 243.2109, 170.1541, 95.0854
A_{26}	16- hydroxyl-octadecanamide	51.30	$C_{18}H_{37}NO_2$	300.2897	300.2889	-2.451	**283.2632**, 239.2369, 221.2263, 91.0540
A_{27}	N-cyclohexane-(3’-hydroxyl)-tridecane amide	51.97	$C_{20}H_{39}NO_3$	342.3002	342.2996	-0.621	**324.2897**, 239.2369, 221.2261, 109.1010, 104.0705
A_{28}	N-(3,4-dihydroxybenzyl)-(9Z)-tetradecanamide	52.44	$C_{20}H_{39}NO_2$	326.3053	326.3048	-1.673	**309.2786**, 265.2523, 247.2417, 135.1166, 121.1010
A_{29}	N-(3,4-dihydroxy-benzyl)-(9E)-tetradecanamide	52.77	$C_{20}H_{39}NO_2$	326.3053	326.3045	-2.409	**309.2785**, 265.2521, 247.2415, 135.1165, 121.1009
A_{30}	N-benzyl-9-oxo-12N-octadecenamide	52.93	$C_{25}H_{39}NO_2$	386.3053	386.3045	-2.138	369.2789, **351.2681**, 263.2370, 245.2264, 107.0491
A_{31}	N-benzyl-8E, 10E-hexadecadienamide	53.53	$C_{23}H_{35}NO$	342.2791	342.2782	0.941	325.2526, 235.2058, **217.1952**, 190.1228, 91.0541
A_{32}*	N-(3-methoxybenzyl)-(9Z,12Z,15Z)-octadecatrienamide	54.55	$C_{26}H_{39}NO_2$	398.3053	398.3031<br>420.2843	1.126	**381.2788**, 290.2116, 261.2214, 243.2108, 121.0648
A_{33}	N-ethyl-(9E,11E)-octadecadienamide	54.67	$C_{20}H_{37}NO$	308.2947	308.2942	1.756	263.2371, **245.2265**, 238.2167, 109.1011, 95.0854
A_{34}	N-benzyl-(9Z,12Z,15Z)-octadecatrienamide	54.98	$C_{25}H_{37}NO$	368.2947	368.2929<br>390.2743	1.851	**351.2678**, 333.2573, 261.2211, 243.2105, 121.1010

A_{35}	N-benzyl-9Z,12Z-hexadecadienamide	55.98	$C_{24}H_{37}NO$	356.2947	356.2943	-1.267	339.2675, 249.2209, **231.2103**, 218.1535, 91.0539
A_{36}	N-benzyl-tetradeceneamide	56.10	$C_{21}H_{35}NO$	318.2791	318.2786	-0.451	240.2323, **211.2058**, 193.1951, 123.1168, 91.0540
A_{37}	N-(3-methoxybenzyl)-(9Z)-benzylhexadecanamide	56.16	$C_{23}H_{39}NO_2$	362.3053	362.3046	-1.838	268.2634, 254.2477, 239.2369, 137.1324, **107.0490**, 91.9698
A_{38}	N-benzyl)-13-oxo-11E-heptadecenamide	56.73	$C_{24}H_{39}NO_2$	374.3053	374.3049	-0.978	357.2782, 266.2474, 248.1641, 138.0911, **121.0646**
A_{39}	N- benzyl-9E,12E- hexadecadienamide	56.83	$C_{24}H_{37}NO$	356.2947	356.2941	-1.940	339.2677, 309.2977, 249.2210, **231.2104**, 218.1380, 116.0704, 91.0539
A_{40}	N-benzyl-16-hydroxy-9-oxo-13E,15E-octadecadienamide	56.92	$C_{25}H_{41}NO_2$	388.3210	388.3203	-1.715	371.2937, 280.2630, 265.2522, 247.2417, **124.0755**, 107.0489
A_{41}	N-benzyl-4E-hexadecenamide	57.21	$C_{23}H_{37}NO$	344.2947	344.2944	-0.301	288.2322, 274.2165, 246.1852, **219.2103**, 91.0540
A_{42}	N-benzyl-4Z-hexadecenamide	57.64	$C_{23}H_{37}NO$	344.2947	344.2939	-2.357	288.2317, 274.2161, 246.1848, **219.2103**, 91.0539
A_{43}*	N-(3-methoxybenzyl)-(9Z,12Z)-octadecadienamide	58.14	$C_{26}H_{41}NO_2$	400.3210	400.3210<br>422.3028	-0.026	**383.2948**, 365.2840, 278.2478, 245.2264, 138.0914<br>121.0648
A_{44}	N-octadecanamide	58.39	$C_{18}H_{37}NO$	284.2947	284.2938	-3.206	256.2636, 158.1540, 144.1384, 130.1228, **116.1070**
A_{45}	N-benzyl-(9Z,12Z)-octadecadienamide[29]	58.60	$C_{25}H_{39}NO$	370.3091	370.3091<br>392.2903	-0.595	353.2840,278.2480, 263.2371, **245.2265**, 232.1696, 218.1541
A_{46}	N-benzylpentadecanamide	59.12	$C_{22}H_{37}NO$	332.2533	332.2938	2.833	254.2480, **225.2480**, 207.2107, 137.1325, 123.1168
A_{47}	N-ethyl-(2E,16E)-octadecadienamide	59.12	$C_{20}H_{39}NO$	310.3104	310.3101	-1.036	265.2527, 254.2479, **247.2421**, 95.0854
A_{48}	N-(3,4-dimethoxybenzyl)-hexadecanamide	59.93	$C_{25}H_{43}NO_3$	406.3315	406.3274	-4.091	391.2822, 375.2487, 305.0555, **91.5041**
A_{49}	N-benzyl-14E,8E-eicosenoicdienamide	60.12	$C_{22}H_{39}NO$	334.3104	334.3099	1.500	292.2634, 278.2477, 238.2165, 196.1696, **182.1537**, 109.1011
A_{50}	N-benzyl-9Z, 12Z-heptadecadienamide	60.24	$C_{24}H_{39}NO$	358.3104	358.3099	-1.483	341.2838, 251.2370, **233.2264**, 218.1540, 91.0540
A_{51}	N-benzyl-9Z,12Z-nonadecadienamide	61.09	$C_{26}H_{41}NO$	384.3260	384.3255	-1.305	**367.2990**, 292.2632, 259.2417, 246.1849, 232.1693
A_{52}*	N-(3-methoxybenzyl)-hexadecanamide	61.49	$C_{24}H_{41}NO_2$	376.3210	376.3199	-2.806	360.0393, 268.2636, 239.2370, 138.0914, **121.0648**
A_{53}	N-(3-methoxybenzyl)-(9E)-octadecenamide	61.95	$C_{26}H_{43}NO_2$	402.3366	402.3347	-4.738	385.3099, 367.2991, 294.2789, 247.2417, **121.0647**
A_{54}*	N-benzylhexadecanamide[29]	62.10	$C_{23}H_{39}NO$	346.3104	346.3096	-1.361	268.2633, **239.2368**, 221.2262, 137.1324, 109.1011

					368.2912		
A_{55}	N-(3-methoxybenzyl)-(9Z)-octadecenamide	62.23	$C_{26}H_{43}NO_2$	402.3366	402.3360	-1.631	385.3103, 265.2526, 247.2421, 138.0914, **121.0648**
A_{56}	N-benzyl-9Z-octadecenamide[29]	62.53	$C_{25}H_{41}NO$	372.3260	372.3249	-3.066	355.2995, 294.2792, 265.2527, **247.2422**, 135.1168
A_{57}	N-benzyl-10E,12E-twentycarbondienamide	63.46	$C_{27}H_{43}NO$	398.3417	398.34125	1.234	**381.3150**, 316.2631, 306.2790, 302.2476, 273.2576
A_{58}	N-benzylheptadecanamide[29]	63.83	$C_{24}H_{41}NO$	360.3260	360.3256	-1.225	282.2792, 253.2525, 235.2419, 137.1324, **123.1168**
A_{59}	N-benzyl-12Z-nonadecenamide	64.51	$C_{26}H_{43}NO$	386.3417	386.3410	-1.842	369.2786, **316.2630**, 279.2317, 261.2211, 124.0756, 107.0490
A_{60}	N-benzylheptadecanamide	64.89	$C_{24}H_{41}NO$	360.3260	360.3248	-3.854	282.2787, 253.2522, 235.2416, 137.1322, **123.1165**
A_{61}	N-benzyl-12E-nonadecenamide	65.32	$C_{26}H_{43}NO$	386.3417	386.3408	-2.230	369.2786, 279.2317, **261.2211**, 124.0756, 107.0490
A_{62}*	N-benzyloctadecanamide	67.22	$C_{25}H_{43}NO$	374.3417	374.3414	-0.772	296.3171, 267.2619, 249.2857, 137.1380, **123.1394**
A_{63}	N-benzyloctadecanamide	68.33	$C_{25}H_{43}NO$	374.3417	374.3404	-4.252	296.2946, 267.2681, 249.2573, 137.1323,**123.1166**
A_{64}	N -benzyltwentycarboxamide	78.12	$C_{27}H_{47}NO$	402.3730	402.3704	-0.632	324.3243, 295.2979, 277.2874, 137.1316, **123.1160**

*Structures confimed by comparison with reference standards. Bold characters: the base peaks in MS^n spectra

Table S2 Identification of Macaridines from Maca by UHPLC-ESI-Orbitrap MS

NO	name	t_R(min)	Formula	ESI(+)/expected (*m/z*)	d ESI(+)/measure (*m/z*)	Delta (ppm)	Fragamentor information
A_{65}	3-benzyl-1,2-dihydro-N-hydroxypyridine-4-methoxy	24.33	$C_{13}H_{15}NO_2$	218.1175	218.1174	-0.437	200.1279, 159.3215, 126.0550, 112.0756, **91.0541**
A_{66}*	3-benzyl-1,2-dihydro-N-hydroxypyridine-4-carbaldehyde	27.12	$C_{13}H_{13}NO_2$	216.1019	216.1018	-0.302	**198.0915**, 188.1071, 170.0965, 158.0965, 91.0541
A_{67}*	(3-methoxybenzyl)–N-pyridine-4-carbaldehyde	27.56	$C_{14}H_{13}NO_2$	228.1019	228.1012	-3.048	**200.1072**, 169.0887, 132.4082, 121.0649

*Structures confimed by comparison with reference standards. Bold characters: the base peaks in MS^n spectra

Table S3 Identification of Imidazole alkaloids from Maca by UHPLC-ESI-Orbitrap MS

NO	name	t_R(min)	Formula	ESI(+)/expected (*m/z*)	ESI(+)/measured (*m/z*)	Delta (ppm)	Fragamentor information
A_{68}	1-dibenzyllimidazilium	15.70	$C_8H_{12}N_2$	137.1073	137.1073	0.255	124.4250, **109.0646**, 91.0538, 77.9985
A_{69}	1-dibenzyl-4,5-dimethylimidazilium	19.46	$C_{12}H_{14}N_2$	187.1229	187.1229	-0.187	159.0803, 109.0759, **91.0541**
A_{70}	1-dibenzyl-trimethylimidazilium	20.49	$C_{13}H_{16}N_2$	201.1386	201.1386	0.065	160.1120, 121.1435, 109.0760, **91.0541**
A_{71}	1-dibenzyl-2-ethyl-4,5-dimethylimidazilium	20.91	$C_{14}H_{18}N_2$	215.1542	215.1542	0.002	187.1230, 137.1073, 123.0917, 109.0761, **91.0541**
A_{72}	1-dibenzyl-2-propane-4,5-dimethylimidazilium	21.12	$C_{15}H_{18}N_2$	227.1542	227.1543	0.109	**135.0917**, 91.0540
A_{73}	1-dibenzyl-2-propyne-4,5-dimethylimidazilium	21.82	$C_{15}H_{16}N_2$	225.1386	225.1387	0.333	208.1120, 181.1011, 130.0651, **91.0541**
A_{74}	1-dibenzyl-2-propane-4,5-dimethylimidazilium	21.94	$C_{15}H_{20}N_2$	229.1699	229.1696	-1.026	188.1433, **137.1073**, 121.6014, 91.0540
A_{75}	1-dibenzyl-2-butene-4,5-dimethylimidaziliumc	22.41	$C_{16}H_{20}N_2$	241.1699	241.1698	-0.270	**149.1072**, 121.0653, 91.0539
A_{76}	1-dibenzyl-2-isopropane-4,5-dimethylimidazilium	23.37	$C_{15}H_{20}N_2$	229.1699	229.1696	-1.201	187.1229, 160.1119, **137.1073**, 123.0915, 91.0540
A_{77}	1-dibenzyl-2-(1,3-butadiene)-4,5-dimethylimidazilium	23.53	$C_{16}H_{18}N_2$	239.1542	239.1542	0.005	198.1278, 181.1013, 158.7507, **91.0541**
A_{78}	1-dibenzyl-2-butyl-4,5-dimethylimidazilium	25.10	$C_{16}H_{22}N_2$	243.1855	243.1855	-0.104	**187.1229**, 160.1118, 151.1229, 91.0541
A_{79}	1-dibenzyl-2-phenyl-4,5-dimethylimidazilium	25.45	$C_{18}H_{18}N_2$	263.1542	263.1543	0.208	185.1073, **171.0917**, 160.1120, 144.1019, 91.0541
A_{80}	1-dibenzyl-2-isobutyl-4,5-dimethylimidazilium	25.64	$C_{16}H_{22}N_2$	243.1855	243.1851	-1.667	**187.1228**, 160.1119, 151.1228, 91.0539
A_{81}	1-dibenzyl-2-(2-pentynylalkenyl)-4,5-dimethylimidazilium	25.93	$C_{17}H_{18}N_2$	251.1542	251.1541	-0.105	234.1126, 187.0754, 158.0966, 132.0809, **91.0541**
A_{82}	1,3-dibenzyl-4,5-dimethylimidazolium	26.69	$C_{19}H_{20}N_2$	277.1699	277.1691	-2.761	262.1521, 199.1231, **185.1075**, 109.0760, 91.0541
A_{83}	1,3-dibenzyl-2,4,5-trimethylimidazilium	27.38	$C_{20}H_{22}N_2$	291.1855	291.1849	-2.285	250.1593, 199.1233, 158.0965, 117.0698, **91.0542**
A_{84}	1,3-dibenzyl-2(R)-ethyl-4,5-dimethylimidazilium	28.84	$C_{21}H_{24}N_2$	305.2012	305.2011	-0.280	250.1593, **213.1386**, 185.1074, 123.0917, 91.0542
A_{85}	1,3-dibenzyl-2-(1,3-diacetylene)-4,5-dimethylimidazilium	29.48	$C_{23}H_{20}N_2$	325.1699	325.1697	-0.155	277.2164, 201.1026, 158.0965, 121.0649, **91.0541**
A_{86}	1,3-dibenzyl-2-vinyl-4,5-dimethylimidazilium	29.64	$C_{21}H_{22}N_2$	303.1855	303.1855	-0.182	211.1228, **185.1073**, 173.1071, 131.0854, 91.0540

A_{87}	1,3-dibenzyl-2(S)-ethyl-4,5-dimethylimidazilium	30.03	$C_{21}H_{24}N_2$	305.2012	305.2011	-0.280	**213.1389**, 185.1075, 137.1075, 91.0542
A_{88}	1-dibenzyl-2-heptyl-4,5-dimethylimidazilium	30.03	$C_{19}H_{28}N_2$	285.2325	285.2323	-0.790	267.1495, 193.1701, 138.1152, **91.0541**
A_{89}	1,3-dibenzyl-2-propyl-4,5-dimethylimidazilium	30.25	$C_{22}H_{26}N_2$	319.2168	319.2166	-1.301	**227.1543**, 200.1310, 185.1075, 158.0965, 137.1074, 91.0541
A_{90}	1,3-dibenzyl-2(1,3-glutaricalkynyl)-dimethylimidazilium	30.28	$C_{24}H_{22}N_2$	339.1855	339.1385	-0.458	261.1385, **247.1230**, 181.1011, 158.0961, 91.0542
A_{91}	1,3-dibenzyl-2-isopropyl-4,5-dimethylimidaziliumchloride	30.50	$C_{22}H_{26}N_2$	319.2168	319.2166	-1.301	278.1904, **227.1544**, 200.1309, 181.1012, 91.0541
A_{92}	1-dibenzyl-2-isoheptyl-4,5-dimethylimidazilium	30.75	$C_{19}H_{28}N_2$	285.2325	285.2320	-1.632	268.1331, 204.1212, 160.0756, 121.0647, **91.0540**
A_{93}	1,3-dibenzyl-2-propenyl-4,5-dimethylimidazilium	30.75	$C_{22}H_{24}N_2$	317.2012	317.2010	-0.205	308.7192, 181.1011, 158.0964, 135.0916, **131.0855**, 109.0759, 91.0540
A_{94}	1,3-dibenzyl-2-phenyl-4,5-dimethylimidazilium	31.00	$C_{25}H_{24}N_2$	353.2012	353.2007	-1.261	275.1544, **185.1073**, 171.0917, 158.0965, 121.0653, 91.0541
A_{95}	1,3-dibenzyl-2-butyl-4,5-dimethylimidazilium	31.34	$C_{23}H_{28}N_2$	333.2325	333.2321	-1.217	277.1698, 250.1591, **241.1699**, 200.1309, 185.1073, 158.0963, 91.0522
A_{96}	1,3-dibenzyl-2-isobutyl-4,5-dimethylimidazilium	31.69	$C_{23}H_{28}N_2$	333.2325	333.2324	-0.136	277.1700, 250.1591, **241.1700**, 200.1310, 185.1074, 158.0964, 91.0521
A_{97}	1-dibenzyl-2-(5-en-1,3-heptadiyne)-4,5-dimethylimidazilium	32.91	$C_{26}H_{24}N_2$	365.2012	365.2009	-0.809	310.7103, 287.1543, **185.1074**, 91.6150
A_{98}	1,3-dibenzyl-2-(5-en-1,3-propylene alkyne)-4,5-dimethylimidazilium	32.98	$C_{27}H_{26}N_2$	379.2168	379.2164	-1.016	**288.1619**, 273.1383, 256.1886, 199.1228, 178.0775
A_{99}	1,3-dibenzyl-2-pentyl-4,5-dimethylimidazilium	33.17	$C_{24}H_{30}N_2$	347.2481	347.2479	-0.186	277.1702, **255.1857**, 227.1544, 200.1310, 158.0965, 91.0524
A_{100}	1,3-dibenzyl-2-(3,5,7-trienepropyne) -4,5-dimethylimidazilium	33.32	$C_{27}H_{26}N_2$	379.2168	379.2169	0.118	**288.1619**, 273.1383, 256.1886, 199.1228, 115.0540
A_{101}	1-(3,5-cyclohexadiene)-cyclohexyl-2-hexyl-4,5-dimethyhydridelimidazilium	33.48	$C_{21}H_{35}N_2$	316.2873	316.2842	-3.962	**298.2738**, 280.2633, 262.2525, 109.1010, 91.0541
A_{102}	1-dibenzyl-3-cyclohexyl-2-isopropenyl-4,5-dimethylimidazilium	33.48	$C_{21}H_{35}N_2$	316.2846	316.2843	-1.487	**298.2738**, 280.2633, 245.2261, 109.1010
A_{103}	1-(1,3-cyclohexadiene)-3-cyclohexyl-2-hexyl-4,5-dimethyhydridelimidazilium	33.93	$C_{21}H_{35}N_2$	316.2873	316.2840	-3.211	**298.2739**, 280.2634, 262.2527, 109.1012, 91.0541
A_{104}	1-dibenzyl-3-cyclohexyl-2-propenyl-4,5-dimeth	33.93	$C_{21}H_{35}N_2$	316.2846	316.2841	-2.962	**298.2739**, 280.2633, 245.2260, 135.1166, 109.1009

	ylimidazilium						
A_{105}	1,3-dibenzyl-2-hexyl-4,5-dimethylimidazilium	34.65	$C_{25}H_{32}N_2$	361.2638	361.2637	-0.348	269.2014, 250.1591, 213.1387, 200.1309, **185.1073**, 123.0916, 109.0759, 91.0517

*Structures confimed by comparison with reference standards. Bold characters: the base peaks in MS^n spectra

Table S4 Identification of β-carboline alkaloids from Maca by UHPLC-ESI-Orbitrp MS

NO	name	t_R(min)	Formula	ESI(+)/expected (*m/z*)	ESI(+)/measured (*m/z*)	Delta (ppm)	Fragamentor information
A_{106}	(1R,3S)-1-methyltetrahydro-β-5,6-hydride carboline-3-carboxylic acid	14.71	$C_{13}H_{16}N_2O_2$	233.1284	233.1283	-0.490	215.1176, **116.0705**, 91.0539, 70.0648
A_{107}	(3S)-tetrahydro-β-4,5-hydridecarboline-2-carboxylic acid	15.08	$C_{11}H_{12}N_2O_2$	205.0971	205.0968	-1.727	**188.0708**, 159.0917, 146.0600, 132.0807, 112.0667
A_{108}	(1S,3S)-1-methyltetrahydro-β-5,6-hydride carboline-3-carboxylic acid	16.82	$C_{13}H_{16}N_2O_2$	233.1284	233.1284	0.110	215.1178, 187.1229, 142.0862, **116.0706**, 91.0540
A_{109}	(1R,3S)-tetrahydro-β-5,6-hydridecarboline-3-carboxylic acid	17.08	$C_{12}H_{12}N_2O_2$	217.0971	217.0970	-0.572	200.0706, 171.0917, **144.0808**, 121.0673, 91.0540
A_{110}	(1R,3S)-1-methyltetrahydro-β-carboline-3-carboxylic acid	17.56	$C_{13}H_{14}N_2O_2$	231.1128	231.1126	-0.711	**214.0863**,188.0707, 158.0965, 144.0808, 130.0652 91.0540
A_{111}	(1R,3S)-1-ethyltetrahydro-β-5,6-carboline-3-carboxylic acid	18.21	$C_{14}H_{16}N_2O_2$	245.1284	245.1284	-0.099	228.1021, 199.1231, **153.0659**, 138.1107, 121.0648, 109.0760, **91.0541**
A_{112}	(1R,3S)-1-butanetetrahydro-β-5,6-hydride carboline-3-carboxylic acid	19.02	$C_{16}H_{18}N_2O_2$	271.1441	271.1436	-1.786	252.1226, **179.0814**, 135.0915, 91.0540
A_{113}	(1R,3S)-1-pentanetetrahydro-β-5,6-carboline-3-carboxylic acid	19.59	$C_{17}H_{20}N_2O_2$	285.1597	285.1597	0.125	267.1493, 239.1544, 193.0973,**177.1024**, 135.0918 91.0541
A_{114}	(1R,3S)-1-butanetetrahydro-β-5,6-hydride carboline-3-carboxylic acid	20.17	$C_{16}H_{20}N_2O_2$	273.1597	273.1597	0.130	255.1492, **187.1230**, 121.0646, 109.0759, 91.0541
A_{115}	(1R,3S)-1-pentanetetrahydro-β-5,6-hydride carboline-3-carboxylic acid	21.06	$C_{17}H_{22}N_2O_2$	287.1754	287.1750	-1.130	246.1486, **201.1386**, 195.1127, 109.0758, 91.0539
A_{116}	(1R,3S)-1-(2-methylbutane)tetrahydro-β-5,6-hydride carboline-3-carboxylic acid	22.73	$C_{17}H_{22}N_2O_2$	287.1754	287.1749	-1.443	241.1698, **195.1127**, 187.1229, 109.0759, 91.0540

A_{117}	(1R,3S)-1-hexyltetrahydro-β-5,6-hydride carboline-3-carboxylic acid	23.73	$C_{18}H_{24}N_2O_2$	301.1910	301.1908	-0.546	**283.1804**, 260.1642, 209.1285, 121.0647, 91.0540
A_{118}	(1R,3S)-1-(3-methylpentane)tetrahydro-β-5,6-hydridecarboline-3-carboxylic acid	24.62	$C_{18}H_{24}N_2O_2$	301.1910	301.1908	-0.546	255.1853, 209.1284, **187.1229**, 121.0646, 91.0540
A_{119}	(1R,3S)-1-(1,2-dimethylbutane)tetrahydro-β-5,6-hydride carboline-3-carboxylic acid	24.90	$C_{18}H_{24}N_2O_2$	301.1910	301.1907	-1.177	255.1853, 209.1283, **187.1229**, 109.0757, 91.0540
A_{120}	(1R,3S)-1-isobutanetetrahydro-β-5,6-hydride carboline-3-carboxylic acid	30.47	$C_{16}H_{18}N_2O_2$	271.1441	271.1438	-0.791	211.1118, 138.0913, 121.0647, 108.0807, **91.0540**
A_{121}^{*}	(1R,3S)-1-methyl-β-carboline-3-carbaldehyde	32.59	$C_{13}H_{10}N_2O$	211.0865	211.0865	-0.140	193.07689, **185.0716**, 169.0767

*Structures confimed by comparison with reference standards. Bold characters: the base peaks in MS^n spectra

Table S5 Identification of Acids from Maca by UHPLC-ESI-Orbitrap MS

NO	name	t_R(min)	Formula	ESI(-)/expected (*m/z*)	ESI(-)/measured (*m/z*)	Delta (ppm)	Fragamentor information
C_1^{*}	3-methoxyphenylacetic acid	2.17	$C_9H_{10}O_3$	165.0462	165.0403	0.184	147.0304, **129.0197**, 75.0091
C_2^{*}	2,4-dihydroxy-3,5-cyclopentyl dienoic acid	2.33	$C_6H_7O_4$	143.0338	143.0333	-3.812	**69.0328**
C_3^{*}	Nicotinic acid	3.98	$C_6H_5NO_2$	122.0245	122.0245	0.007	102.2594, **78.0348**, 66.0872, 53.6929
C_4^{*}	4-hydroxy-3-methoxybenzoicacid	5.31	$C_8H_8O_4$	167.0339	167.0203	-0.979	**152.0114**, 123.0451, 108.0217, 95.0137
C_5^{*}	Succinic acid	6.80	$C_4H_6O_4$	117.0182	117.0189	4.280	**99.0088**, 73.0295

Table S6 Identification of Glucosinolates from Maca by UHPLC-ESI-Orbitrap MS

NO	name	t_R(min)	Formula	ESI(-)/expected (*m/z*)	ESI(-)/measured (*m/z*)	Delta (ppm)	Fragamentor information
G_1	indolyl-3-methylglucosinolate	10.65	$C_{15}H_{29}O_{10}NS_2$	446.1154	446.1115	-3.859	424.0449, 383.1183, **274.9914**, 249.9780, 164.0169
G_2	indolyl-5-methylglucosinolate	11.53	$C_{15}H_{29}O_{10}NS_2$	446.1154	446.1116	-3.658	424.0466, 383.1354, **274.9914**, 249.9784, 164.0171
G_3^{*}	m-methoxybenzylglucosinolate	11.99	$C_{15}H_{20}O_{10}NS_2$	438.0523	438.0503	-4.551	358.0956, 274.9897, **259.0126**, 242.0125, 227.0225, 196.0435, 163.0609

G_4	p-methoxybenzylglucosinolate	12.89	$C_{15}H_{20}O_{10}NS_2$	438.0523	438.0508	-4.616	358.0957, 274.9898, **259.0127**, 242.0127, 227.0226, 196.0437, 163.0610
G_5*	benzylglucosinolate	14.96	$C_{14}H_{19}O_9NS_2$	408.0423	408.0399	-4.679	328.0852, 274.9896, **259.0124**, 241.0020, 230.0123 , 212.0020, 195.0328, 166.0331
G_6	benzylglucosinolate	15.61	$C_{14}H_{19}O_9NS_2$	408.0423	408.0402	-4.091	328.0853, 274.9898, **259.0124**, 241.0022, 230.0124 , 212.0021, 195.0329, 166.0332
G_7	th-hydroxybenzylglucosinolate	17.12	$C_{14}H_{19}O_{10}NS_2$	424.0372	424.0350<br>446.0165	-4.216	334.1130, 290.9841, 274.9896, **259.0125**, 227.9968<br>182.0279, 148.0402
G_8*	p-hydroxybenzylglucosinolate	17.79	$C_{14}H_{19}O_{10}NS_2$	424.0372	424.0347<br>446.0158	-4.853	344.0798, 290.9841, 274.9894, **259.0123**, 227.9967,<br>182.0278, 148.0402
G_9	4-methoxyindolyl-3-methyloxyglucosinolate	20.61	$C_{17}H_{22}O_{10}N_2S_2$	477.0632	477.0611	-3.737	431.1538, 408.0408, 360.7300, 274.9891, **259.0120**<br>181.0544, 163.0608, 138.9703
G_{10}	4-methoxyindolyl-3-hexylhydroxyglucosinolate	20.94	$C_{22}H_{36}O_{10}N_2S_2$	551.1727	551.1728	0.720	491.1543, 341.1025, **235.0608**, 178.0269, 165.0555
G_{11}	acetyl-benzylglucosinolate	21.30	$C_{16}H_{21}O_{10}NS_2$	450.0523	450.0505	-3.874	390.0305, 370.0950, 316.9993, **301.0225**, 230.0120,<br>212.0016, 166.0327
G_{12}	acetyl-benzylglucosinolate	21.89	$C_{16}H_{21}O_{10}NS_2$	450.0523	450.0512	-2.386	390.0309, 370.0951, 316.9999, **301.0230**, 230.0122,<br>212.0020, 166.0329
G_{13}	Pent-4-enylglucosinolate	22.92	$C_{12}H_{24}O_7N_2S_2$	371.0941	371.0964	4.174	353.0862, 311.0759, **249.0612**, 231.0503, 175.0242, 121.0292
G_{14}	indolyl3-hexyl-4-methyl-cyclohexaneglucosinolate	24.76	$C_{22}H_{38}O_9N_2S_2$	521.1985	521.1992	1.220	401.1591, 371.1486, **359.1495**, 178.0633, 161.0453

*Structures confimed by comparison with reference standards. Bold characters: the base peaks in MS^n spectra

Table S7 Identification of Macaenes from Maca by UHPLC-ESI-Orbitrap MS

NO	name	t_R(min)	Formula	ESI(+)/expected (*m/z*)	ESI(+)/measured (*m/z*)	Delta (ppm)	Fragamentor information
M_1	5-oxo-6E,8E,10E,12E-octadecatetraenoic acid	32.49	$C_{18}H_{26}O_3$	291.1954	291.1952	-0.863	**273.1852**, 255.1746, 237.1639, 107.0855, 97.1011
M_2	5-oxo-6E,8E,10E,12E,14E-octadecapentaenoic acid	34.68	$C_{18}H_{24}O_3$	289.1798	289.1797	-0.142	**271.1695**, 253.1590, 233.1538, 215.1432, 91.0541

M_3	5-oxo-6E,8E,10E-octadecatrienoic acid	34.77	$C_{18}H_{28}O_3$	293.2111	293.2111	0.200	**275.2007**, 257.1899, 201.1025, 121.0648, 91.0541
M_4	5-oxo-6E,8E,10E,12E,14E,16E,18E,20E,22E-twentysixcarbonnonaenoic acid	38.31	$C_{26}H_{32}O_3$	393.2424	393.2394	-2.505	**376.3320**, 239.2370, 221.2263, 123.1168
M_5	5-oxo-6E,8E,10E,12E-eicosatetraenoic acid	40.71	$C_{20}H_{30}O_3$	319.2267	319.2263	-1.320	301.2168, **273.1854**, 255.1748, 237.1642, 153.0913, 135.0807, 107.0856
M_6	5-oxo-6E,8E,10E,12E,14E,16E,18E,20E,22E,24E,26E -octacosundecenoic acid	43.13	$C_{28}H_{32}O_3$	417.24242	417.2393	-2.289	**389.2587**, 361.1774, 293.1148, 189.1638, 123.1167
M_7	5-oxo-6E,8E-octadecadienoic acid	45.39	$C_{18}H_{30}O_3$	295.2267	295.2262	-1.630	**277.2165**, 259.2059, 241.1953, 135.1169, 95.0854
M_8	5-oxo-6E,8E,10E-eicosatrienoic acid	52.01	$C_{20}H_{32}O_3$	321.2424	321.2417<br>343.2234	-1.965	303.2323, 285.2215, **275.2004**, 257.1904, 153.0912, 135.0806
M_9	5-oxo-6E,8E-nineteencarbodienoic acid	53.02	$C_{19}H_{32}O_3$	309.2424	309.2417<br>331.2235	-2.236	**277.2164**, 259.2058, 185.1170, 153.0911, 107.0855
M_{10}	5-oxo-6E,8E,10E,12E,14E,16E,18E-eicosatanabatienoic acid	55.50	$C_{20}H_{24}O_3$	323.2580	323.2572<br>345.2391	-0.841	305.2480, **277.2168**, 259.2061, 153.0913, 135.0807 107.0856
M_{11}	5-oxo-6E- eicosanoic acid	57.08	$C_{20}H_{36}O_3$	325.2737	325.2734	-0.773	308.2977, **277.2166**, 239.2009, 153.0912, 135.0806, 107.0856

*Structures confimed by comparison with reference standards. Bold characters: the base peaks in MS^n spectra

Table S8 Idetification of Other compounds from Maca by UHPLC-ESI-Orbitrap MS

NO	name	t_R(min)	Formula	ESI(+)/expected (*m/z*)	ESI(+)/measured (*m/z*)	Delta (ppm)	Fragamentor information
O_1*	2-furoate methyl ester	2.98	$C_6H_6O_3$	127.0389	127.0387	-1.500	**95.0488**, 68.4856
O_2*	1-formaldehyde-2-hydroxy-5-methoxypyridine	10.22	$C_7H_7NO_3$	154.0497	154.0498	0.160	205.0852, **195.9230**, 149.0227
O_3	N-(3-hydroxy-5-methyl-benzyl)-2Z-2-amino-decatrienamide	15.08	$C_{11}H_{12}N_2O_2$	205.0971	205.0968	-1.727	190.0773, **188.0709**, 170.0598, 121.0674, 91.0546
O_4*	3-hydroxybenzylcyanide	19.15	C_8H_7NO	134.0600	134.0593	-4.001	**107.0485**
O_5*	catechin	27.15	$C_{15}H_{14}O_6$	291.0863	291.0867	1.530	273.0766, **165.0550**, 151.0394, 139.0394, 123.0444

O_6	Licochalcone A	30.13	$C_{21}H_{22}O_4$	339.1592	339.1591	-0.278	**297.1497**, 283.0977, 271.0977, 245.1182, 121.0289
O_7	N-ethyl-tetradecene ester	31.69	$C_{16}H_{35}NO_2$	274.2740	274.2740	-0.021	**256.2637**, 212.2373, 136.1121, 93.4281
O_8^*	2,3-dihydroxy-acetic acid methyl ester	33.22	$C_9H_9O_4$	182.0573	182.0623	2.132	104.0158, **91.0536**, 78.6403, 76.0209
O_9^*	dibutyl phthalate	47.22	$C_{16}H_{22}O_4$	279.1579<br>301.1396	279.1583	2.247	**261.2211**, 205.0864, 149.0235

*Structures confimed by comparison with reference standards. Bold characters: the base peaks in MS^n spectra

Table S9 Precision, stability and repeatability of 15 compounds

Compounds	Precision (RSD%, n=3)		Stability (RSD%, n=6)	Repeatibility (RSD%, n=6)
	Intra day	Inter day		
G_5	2.46	0.43	2.22	1.97
G_3	1.81	0.86	2.58	1.05
G_8	3.11	1.28	2.33	2.78
A_{52}	1.73	0.80	2.63	2.75
A_8	1.56	0.66	1.82	2.33
A_{54}	0.98	1.65	1.86	2.06
O_6	1.30	0.99	1.98	1.01
A_{66}	0.63	1.83	1.41	2.63
A_{67}	1.73	1.19	2.22	2.34
A_5	0.41	1.44	2.39	2.03
A_4	1.49	0.88	2.57	2.84
C_3	2.36	2.71	1.85	2.71

C_5	1.41	1.26	1.02	1.18
A_{121}	2.02	1.19	1.93	2.27
O_9	1.64	0.76	1.73	1.06

Table S10 Recoveries of 15 constituents (n=9)

Compound	Addition level	Initial / μg	Added/ μg	Measured/ μg	Recovery rate /%	Average/%	RSD/%
	1:0.8	36957.84	29566.26	65509.98	96.57		
G_5	1:1	36957.84	36957.85	73302.19	98.34	97.60	0.94
	1:1.2	36957.84	44349.40	80367.03	97.88		
	1:0.8	1421.32	1137.05	2537.79	98.19		
G_3	1:1	1421.32	1421.30	2828.83	99.03	97.96	1.22
	1:1.2	1421.32	1705.59	3070.11	96.67		
	1:0.8	369.62	295.71	665.48	100.05		
G_8	1:1	369.62	369.58	733.14	98.36	98.89	1.01
	1:1.2	369.62	443.55	805.50	98.27		
	1:0.8	930.86	744.70	1652.33	96.88		
A_{52}	1:1	930.86	930.83	1820.64	95.59	96.95	1.44
	1:1.2	930.86	1117.05	2029.70	98.37		
	1:0.8	12.73	10.20	22.95	100.20		
A_8	1:1	12.73	12.70	24.97	96.38	98.73	2.08
	1:1.2	12.73	15.28	27.95	99.61		
A_{54}	1:0.8	431.74	345.40	767.64	97.25	98.68	1.38
	1:1	431.74	431.69	863.26	99.96		

	1:1.2	431.74	518.10	943.78	98.83		
	1:0.8	0.39	0.32	0.70	96.88		
O_6	1:1	0.39	0.31	0.69	96.77	96.49	0.60
	1:1.2	0.39	0.48	0.85	95.83		
	1:0.8	8.56	6.85	15.16	96.35		
A_{66}	1:1	8.56	8.53	16.86	97.30	96.39	0.92
	1:1.2	8.56	10.28	18.38	95.53		
	1:0.8	2.45	1.98	4.40	98.48		
A_{67}	1:1	2.45	2.44	4.87	99.18	97.98	1.55
	1:1.2	2.45	2.95	5.29	96.27		
	1:0.8	19.93	15.95	35.91	100.19		
A_5	1:1	19.93	19.89	39.03	96.03	98.36	2.16
	1:1.2	19.93	23.95	43.61	98.87		
	1:0.8	4.61	3.69	8.11	94.85		
A_4	1:1	4.61	4.59	9.03	96.30	96.27	1.46
	1:1.2	4.61	5.55	10.03	97.66		
	1:0.8	10.17	8.15	18.02	96.32		
C_3	1:1	10.17	10.15	20.15	98.33	97.15	1.08
	1:1.2	10.17	12.20	21.98	96.80		
	1:0.8	73.04	58.45	130.45	98.22		
C_5	1:1	73.04	73.08	145.43	99.06	98.07	1.10
	1:1.2	73.04	87.65	157.99	96.92		
A_{121}	1:0.8	0.21	0.17	0.37	94.12	96.71	3.11

	1:1	0.21	0.21	0.42	100.00		
	1:1.2	0.21	0.25	0.45	96.00		
	1:0.8	2.89	2.31	5.18	99.13		
O_9	1:1	2.89	2.90	5.72	97.59	97.66	1.47
	1:1.2	2.89	3.48	6.24	96.26		

Table S11 Samples of Maca from different places

NO.	Places	Weight（g）
1	Xingjiang-1	1.15
2	Xinjiang-2	1.07
3	Xizang-1	1.03
4	Xizang-2	1.03
5	Peru-1	1.16
6	Peru-2	1.02
7	Yunnan-1	1.03
8	Yunnan-2	1.08
9	Yunnan-3	1.02
10	Yunnan-4	1.04
11	Yunnan-5	1.06
12	Yunnan-6	1.06
13	Yunnan-7	1.05
14	Yunnan-8	1.07
15	Yunnan-9	1.12
16	Yunnan-10	1.07
17	Yunnan-11	1.04
Leaves of Maca (12)	Yunnan-6	1.04
